# Quantum machine learning beyond kernel methods

**DOI:** 10.1038/s41467-023-36159-y

**Published:** 2023-01-31

**Authors:** Sofiene Jerbi, Lukas J. Fiderer, Hendrik Poulsen Nautrup, Jonas M. Kübler, Hans J. Briegel, Vedran Dunjko

**Affiliations:** 1 Institute for Theoretical Physics, University of Innsbruck, Technikerstr. 21a, A-6020 Innsbruck, Austria; 2 Max Planck Institute for Intelligent Systems, Tübingen, Germany; 3 Leiden University, Niels Bohrweg 1, 2333 CA Leiden, The Netherlands

**Keywords:** Quantum information, Computer science

## Abstract

Machine learning algorithms based on parametrized quantum circuits are prime candidates for near-term applications on noisy quantum computers. In this direction, various types of quantum machine learning models have been introduced and studied extensively. Yet, our understanding of how these models compare, both mutually and to classical models, remains limited. In this work, we identify a constructive framework that captures all standard models based on parametrized quantum circuits: that of linear quantum models. In particular, we show using tools from quantum information theory how data re-uploading circuits, an apparent outlier of this framework, can be efficiently mapped into the simpler picture of linear models in quantum Hilbert spaces. Furthermore, we analyze the experimentally-relevant resource requirements of these models in terms of qubit number and amount of data needed to learn. Based on recent results from classical machine learning, we prove that linear quantum models must utilize exponentially more qubits than data re-uploading models in order to solve certain learning tasks, while kernel methods additionally require exponentially more data points. Our results provide a more comprehensive view of quantum machine learning models as well as insights on the compatibility of different models with NISQ constraints.

## Abstract (web summary)

Comparing the capabilities of different quantum machine learning protocols is difficult. Here, the authors show that different learning models based on parametrized quantum circuits can all be seen as quantum linear models, thus driving general conclusions on their resource requirements and capabilities.

## Introduction

In the current noisy intermediate-scale quantum (NISQ) era^1^, a few methods have been proposed to construct useful quantum algorithms that are compatible with mild hardware restrictions^2,3^. Most of these methods involve the specification of a quantum circuit Ansatz, optimized in a classical fashion to solve specific computational tasks. Next to variational quantum eigensolvers in chemistry^4^ and variants of the quantum approximate optimization algorithm^5^, machine learning approaches based on such parametrized quantum circuits^6^ stand as some of the most promising practical applications to yield quantum advantages.

In essence, a supervised machine learning problem often reduces to the task of fitting a parametrized function—also referred to as the machine learning model—to a set of previously labeled points, called a training set. Interestingly, many problems in physics and beyond, from the classification of phases of matter^7^ to predicting the folding structures of proteins^8^, can be phrased as such machine learning tasks. In the domain of quantum machine learning^9,10^, an emerging approach for this type of problem is to use parametrized quantum circuits to define a hypothesis class of functions^11–16^. The hope is for these parametrized models to offer representational power beyond what is possible with classical models, including highly successful deep neural networks. And indeed, we have substantial evidence of such a quantum learning advantage for artificial problems^16–21^, but the next frontier is to show that quantum models can be advantageous in solving real-world problems as well. Yet, it is still unclear which of these models we should preferably use in practical applications. To bring quantum machine learning models forward, we first need a deeper understanding of their learning performance guarantees and the actual resource requirements they entail.

**Fig. 1:**
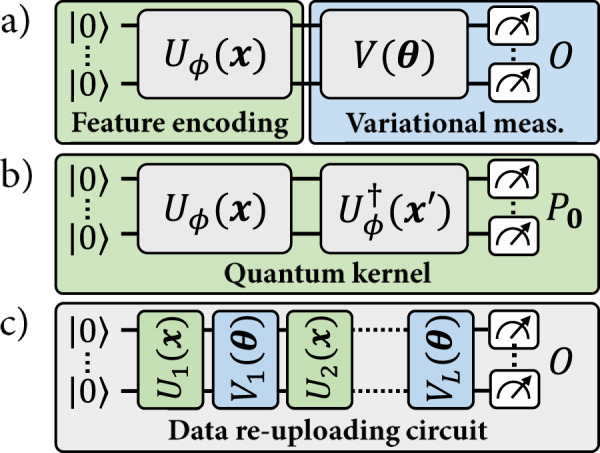
The quantum machine learning models studied in this work. **a** An explicit quantum model, where the label of a data point ***x*** is specified by the expectation value of a variational measurement on its associated quantum feature state *ρ*(***x***). **b** The quantum kernel associated with these quantum feature states. The expectation value of the projection ${P}_{{{{{{{{\mathbf{0}}}}}}}}}=\left|{{{{{{{\mathbf{0}}}}}}}}\right\rangle \left\langle {{{{{{{\mathbf{0}}}}}}}}\right|$ corresponds to the inner product between *ρ*(***x***) and $\rho ({{{{{{{{\boldsymbol{x}}}}}}}}}^{{\prime} })$. An implicit quantum model is defined by a linear combination of such inner products, for ***x*** an input point and ${{{{{{{{\boldsymbol{x}}}}}}}}}^{{\prime} }$ training data points. **c** A data re-uploading model, interlaying data-encoding and variational unitaries before a final measurement.

Previous works have made strides in this direction by exploiting a connection between some quantum models and kernel methods from classical machine learning^22^. Many quantum models indeed operate by encoding data in a high-dimensional Hilbert space and using solely inner products evaluated in this feature space to model the properties of the data. This is also how kernel methods work. Building on this similarity, the authors of refs. ^23,24^ noted that a given quantum encoding can be used to define two types of models (see Fig. 1): (a) explicit quantum models, where an encoded data point is measured according to a variational observable that specifies its label, or (b) implicit kernel models, where weighted inner products of encoded data points are used to assign labels instead. In the quantum machine learning literature, much emphasis has been placed on implicit models^20,25–31^, in part due to a fundamental result known as the representer theorem^22^. This result shows that implicit models can always achieve a smaller labeling error than explicit models, when evaluated on the same training set. Seemingly, this suggests that implicit models are systematically more advantageous than their explicit counterparts in solving machine learning tasks^25^. This idea also inspired a line of research where, in order to evaluate the existence of quantum advantages, classical models were only compared to quantum kernel methods. This restricted comparison led to the conclusion that classical models could be competitive with (or outperform) quantum models, even in tailored quantum problems^20^.

In recent times, there has also been progress in so-called data re-uploading models^32^ which have demonstrated their importance in designing expressive models, both analytically^33^ and empirically^15,16,32^, and proving that (even single-qubit) parametrized quantum circuits are universal function approximators^34,35^. Through their alternation of data-encoding and variational unitaries, data re-uploading models can be seen as a generalization of explicit models. However, this generalization also breaks the correspondence to implicit models, as a given data point ***x*** no longer corresponds to a fixed encoded point *ρ*(***x***). Hence, these observations suggest that data re-uploading models are strictly more general than explicit models and that they are incompatible with the kernel-model paradigm. Until now, it remained an open question whether some advantage could be gained from data re-uploading models, in light of the guarantees of kernel methods.

**Fig. 2:**
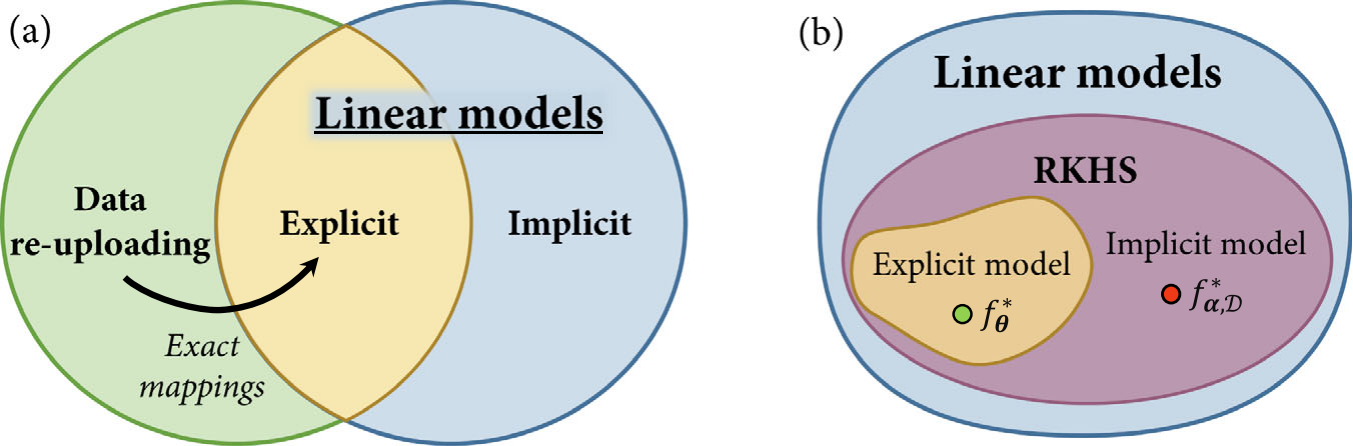
The model families in quantum machine learning. **a** While data re-uploading models are by definition a generalization of linear quantum models, our exact mappings demonstrate that any polynomial-size data re-uploading model can be realized by a polynomial-size explicit linear model. **b** Kernelizing an explicit model corresponds to turning its observable into a linear combination of feature states *ρ*(***x***), for ***x*** in a dataset ${{{{{{{\mathcal{D}}}}}}}}$. The representer theorem guarantees that, for any dataset ${{{{{{{\mathcal{D}}}}}}}}$, the implicit model ${f}_{{{{{{{{\boldsymbol{\alpha }}}}}}}},{{{{{{{\mathcal{D}}}}}}}}}^{*}$ minimizing the training loss associated with ${{{{{{{\mathcal{D}}}}}}}}$ outperforms any explicit minimizer ${f}_{{{{{{{{\boldsymbol{\theta }}}}}}}}}^{*}$ from the same Reproducing Kernel Hilbert Space (RKHS) with respect to this same training loss. However, depending on the feature encoding *ρ*(⋅) and the data distribution, a restricted dataset ${{{{{{{\mathcal{D}}}}}}}}$ may cause the implicit minimizer ${f}_{{{{{{{{\boldsymbol{\alpha }}}}}}}},{{{{{{{\mathcal{D}}}}}}}}}^{*}$ to severely overfit on the dataset and have dramatically worse generalization performance than ${f}_{{{{{{{{\boldsymbol{\theta }}}}}}}}}^{*}$.

In this work, we introduce a unifying framework for explicit, implicit and data re-uploading quantum models (see Fig. 2). We show that all function families stemming from these can be formulated as linear models in suitably defined quantum feature spaces. This allows us to systematically compare explicit and data re-uploading models to their kernel formulations. We find that, while kernel models are guaranteed to achieve a lower training error, this improvement can come at the cost of a poor generalization performance outside the training set. Our results indicate that the advantages of quantum machine learning may lie beyond kernel methods, more specifically in explicit and data re-uploading models. To corroborate this theory, we quantify the resource requirements of these different quantum models in terms of the number of qubits and data points needed to learn. We show the existence of a regression task with exponential separations between each pair of quantum models, demonstrating the practical advantages of explicit models over implicit models, and of data re-uploading models over explicit models. From an experimental perspective, these separations shed light on the resource efficiency of different quantum models, which is of crucial importance for near-term applications in quantum machine learning.

## Results

### A unifying framework for quantum learning models

We start by reviewing the notion of linear quantum models and explain how explicit and implicit models are by definition linear models in quantum feature spaces. We then present data re-uploading models and show how, despite being defined as a generalization of explicit models, they can also be realized by linear models in larger Hilbert spaces.

### Linear quantum models

Let us first understand how explicit and implicit quantum models can both be described as linear quantum models^25,36^. To define both of these models, we first consider a feature encoding unitary ${U}_{\phi }:{{{{{{{\mathcal{X}}}}}}}}\to {{{{{{{\mathcal{F}}}}}}}}$ that maps input vectors ${{{{{{{\boldsymbol{x}}}}}}}}\in {{{{{{{\mathcal{X}}}}}}}}$, e.g., images in ${{\mathbb{R}}}^{d}$, to *n*-qubit quantum states $\rho ({{{{{{{\boldsymbol{x}}}}}}}})={U}_{\phi }({{{{{{{\boldsymbol{x}}}}}}}})\left|{{{{{{{\mathbf{0}}}}}}}}\right\rangle\left\langle {{{{{{{\mathbf{0}}}}}}}}\right|{U}_{\phi }^{{{{\dagger}}} }({{{{{{{\boldsymbol{x}}}}}}}})$ in the Hilbert space ${{{{{{{\mathcal{F}}}}}}}}$ of 2^*n*^ × 2^*n*^ Hermitian operators.

A linear function in the quantum feature space ${{{{{{{\mathcal{F}}}}}}}}$ is defined by the expectation values

1 $$f({{{{{{{\boldsymbol{x}}}}}}}})=\,{{\mbox{Tr}}}\,[\rho ({{{{{{{\boldsymbol{x}}}}}}}})O],$$

for some Hermitian observable $O\in {{{{{{{\mathcal{F}}}}}}}}$. Indeed, one can see from Eq. (1) that *f*(***x***) is the Hilbert–Schmidt inner product between the Hermitian matrices *ρ*(***x***) and *O*, which is by definition a linear function of the form ${\langle \phi ({{{{{{{\boldsymbol{x}}}}}}}}),w\rangle }_{{{{{{{{\mathcal{F}}}}}}}}}$, for *ϕ*(***x***) = *ρ*(***x***) and *w* = *O*. In a regression task, these real-valued expectation values are used directly to define a labeling function, while in a classification task, they are post-processed to produce discrete labels (using, for instance, a sign function).

Explicit and implicit models differ in the way they define the family of observables {*O*} they each consider.

An explicit quantum model^23,24^ using the feature encoding *U*_*ϕ*_(***x***) is defined by a variational family of unitaries *V*(***θ***) and a fixed observable *O*, such that

2 $${f}_{{{{{{{{\boldsymbol{\theta }}}}}}}}}({{{{{{{\boldsymbol{x}}}}}}}})=\,{{\mbox{Tr}}}\,[\rho ({{{{{{{\boldsymbol{x}}}}}}}}){O}_{{{{{{{{\boldsymbol{\theta }}}}}}}}}],$$

for *O*_***θ***_ = *V*(***θ***)^†^*OV*(***θ***), specify its labeling function. Restricting the family of variational observables ${\{{O}_{{{{{{{{\boldsymbol{\theta }}}}}}}}}\}}_{{{{{{{{\boldsymbol{\theta }}}}}}}}}$ is equivalent to restricting the vectors *w* accessible to the linear quantum model $f({{{{{{{\boldsymbol{x}}}}}}}})\,=\,{\langle \phi ({{{{{{{\boldsymbol{x}}}}}}}}),w\rangle }_{{{{{{{{\mathcal{F}}}}}}}}},\,w\,\in \,{{{{{{{\mathcal{F}}}}}}}}$, associated with the encoding *ρ*(***x***).

Implicit quantum models^23,24^ are constructed from the quantum feature states *ρ*(***x***) in a different way. Their definition depends directly on the data points {***x***^(1)^, …, ***x***^(*M*)^} in a given training set ${{{{{{{\mathcal{D}}}}}}}}$, as they take the form of a linear combination

3 $${f}_{{{{{{{{\boldsymbol{\alpha }}}}}}}},{{{{{{{\mathcal{D}}}}}}}}}({{{{{{{\boldsymbol{x}}}}}}}})=\mathop{\sum }\limits_{m=1}^{M}{\alpha }_{m}k({{{{{{{\boldsymbol{x}}}}}}}},{{{{{{{{\boldsymbol{x}}}}}}}}}^{(m)}),$$

for $k({{{{{{{\boldsymbol{x}}}}}}}},{{{{{{{{\boldsymbol{x}}}}}}}}}^{(m)})={\langle \phi ({{{{{{{\boldsymbol{x}}}}}}}}),\phi ({{{{{{{{\boldsymbol{x}}}}}}}}}^{(m)})\rangle }_{{{{{{{{\mathcal{F}}}}}}}}}=\,{{\mbox{Tr}}}\,[\rho ({{{{{{{\boldsymbol{x}}}}}}}})\rho ({{{{{{{{\boldsymbol{x}}}}}}}}}^{(m)})]$ the kernel function associated with the feature encoding *U*_*ϕ*_(***x***). By linearity of the trace, however, we can express any such implicit model as a linear model in ${{{{{{{\mathcal{F}}}}}}}}$, defined by the observable:

4 $${O}_{{{{{{{{\boldsymbol{\alpha }}}}}}}},{{{{{{{\mathcal{D}}}}}}}}}=\mathop{\sum }\limits_{m=1}^{M}{\alpha }_{m}\rho ({{{{{{{{\boldsymbol{x}}}}}}}}}^{(m)}).$$

Therefore, both explicit and implicit quantum models belong to the general family of linear models in the quantum feature space ${{{{{{{\mathcal{F}}}}}}}}$.

### Linear realizations of data re-uploading models

Data re-uploading models^32^ on the other hand do not naturally fit this formulation. These models generalize explicit models by increasing the number of encoding layers *U*_*ℓ*_(***x***), 1 ≤ *ℓ* ≤ *L* (which can be all distinct), and interlaying them with variational unitaries *V*_*ℓ*_(***θ***). This results in expectation-value functions of the form:

5 $${f}_{{{{{{{{\boldsymbol{\theta }}}}}}}}}({{{{{{{\boldsymbol{x}}}}}}}})=\,{{\mbox{Tr}}}\,[{\rho }_{{{{{{{{\boldsymbol{\theta }}}}}}}}}({{{{{{{\boldsymbol{x}}}}}}}}){O}_{{{{{{{{\boldsymbol{\theta }}}}}}}}}],$$

for a variational encoding ${\rho }_{{{{{{{{\boldsymbol{\theta }}}}}}}}}({{{{{{{\boldsymbol{x}}}}}}}})\,=\,U({{{{{{{\boldsymbol{x}}}}}}}},{{{{{{{\boldsymbol{\theta }}}}}}}})\left|{{{{{{{\mathbf{0}}}}}}}}\right\rangle \left\langle {{{{{{{\mathbf{0}}}}}}}}\right|{U}^{{{{\dagger}}} }({{{{{{{\boldsymbol{x}}}}}}}},{{{{{{{\boldsymbol{\theta }}}}}}}})$, where $U({{{{{{{\boldsymbol{x}}}}}}}},{{{{{{{\boldsymbol{\theta }}}}}}}})={U}_{L}({{{{{{{\boldsymbol{x}}}}}}}})\mathop{\prod }\nolimits_{\ell=1}^{L-1}{V}_{\ell }({{{{{{{\boldsymbol{\theta }}}}}}}}){U}_{\ell }({{{{{{{\boldsymbol{x}}}}}}}})$, and a variational observable *O*_***θ***_ = *V*_*L*_(***θ***)^†^*OV*_*L*_(***θ***). Given that the unitaries *U*_*ℓ*_(***x***) and ${V}_{{\ell }^{{\prime} }}({{{{{{{\boldsymbol{\theta }}}}}}}})$ do not commute in general, one cannot straightforwardly gather all trainable gates in a final variational observable ${O}_{{{{{{{{\boldsymbol{\theta }}}}}}}}}^{{\prime} }\in {{{{{{{\mathcal{F}}}}}}}}$ as to obtain a linear model ${\tilde{f}}_{{{{{{{{\boldsymbol{\theta }}}}}}}}}({{{{{{{\boldsymbol{x}}}}}}}})={\langle \phi ({{{{{{{\boldsymbol{x}}}}}}}}),{O}_{{{{{{{{\boldsymbol{\theta }}}}}}}}}^{{\prime} }\rangle }_{{{{{{{{\mathcal{F}}}}}}}}}$ with a fixed quantum feature encoding *ϕ*(***x***). Our first contribution is to show that, by augmenting the dimension of the Hilbert space ${{{{{{{\mathcal{F}}}}}}}}$ (i.e., considering circuits that act on a larger number of qubits), one can construct such explicit linear realizations ${\tilde{f}}_{{{{{{{{\boldsymbol{\theta }}}}}}}}}$ of data re-uploading models. That is, given a family of data re-uploading models ${\{\,{f}_{{{{{{{{\boldsymbol{\theta }}}}}}}}}(\cdot )={{\mbox{Tr}}}[\,{\rho }_{{{{{{{{\boldsymbol{\theta }}}}}}}}}(\cdot ){O}_{{{{{{{{\boldsymbol{\theta }}}}}}}}}]\}}_{{{{{{{{\boldsymbol{\theta }}}}}}}}}$, we can construct an equivalent family of explicit models ${\{\,{\tilde{f}}_{{{{{{{{\boldsymbol{\theta }}}}}}}}}(\cdot )={{\mbox{Tr}}}[{\rho }^{{\prime} }(\cdot ){O}_{{{{{{{{\boldsymbol{\theta }}}}}}}}}^{{\prime} }]\}}_{{{{{{{{\boldsymbol{\theta }}}}}}}}}$ that represents all functions in the original family, along with an efficient procedure to map the former models to the latter.

**Fig. 3:**
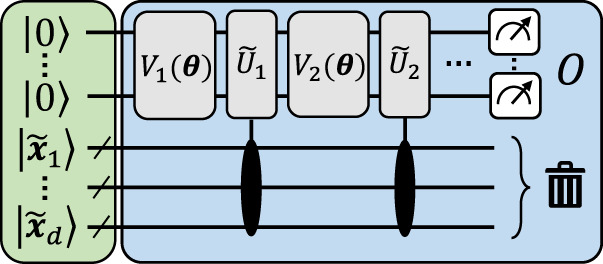
An illustrative explicit model approximating a data re-uploading circuit. The circuit acts *n* working qubits and *dp* encoding qubits. Pauli-X rotations encode bit-string descriptions ${\widetilde{{{{{{{{\boldsymbol{x}}}}}}}}}}_{i}\in {\{0,1\}}^{p}$ of the *d* input components ${x}_{i}\in {\mathbb{R}}$, which constitutes the feature encoding of the explicit model. Fixed and data-independent controlled rotations, interlaid with arbitrary variational unitaries, and a final measurement of the working qubits can result in a good approximation of any parametrized quantum circuit acting on *n* qubits.

Before getting to the main result of this section (Theorem 1), we first present an illustrative construction to convey intuition on how mappings from data re-uploading to explicit models can be realized. This construction, depicted in Fig. 3, leads to approximate mappings, meaning that these only guarantee $|{\,\tilde{f}}_{{{{{{{{\boldsymbol{\theta }}}}}}}}}({{{{{{{\boldsymbol{x}}}}}}}})-{f}_{{{{{{{{\boldsymbol{\theta }}}}}}}}}({{{{{{{\boldsymbol{x}}}}}}}})|\le \,\delta,$ ∀ ***x***, ***θ*** for some (adjustable) error of approximation *δ*. More precisely, we have:

**Proposition 1** *Given an arbitrary data re-uploading model* *f*_***θ***_(***x***) = Tr[*ρ*_***θ***_(***x***)*O*_***θ***_] *as specified by Eq*. (5), *and an approximation error* *δ* > 0, *there exists a mapping that produces an explicit model* ${\tilde{f}}_{{{{{{{{\boldsymbol{\theta }}}}}}}}}({{{{{{{\boldsymbol{x}}}}}}}})=\,{{\mbox{Tr}}}\,[\,{\rho }^{{\prime} }({{{{{{{\boldsymbol{x}}}}}}}}){O}_{{{{{{{{\boldsymbol{\theta }}}}}}}}}^{{\prime} }]$ *as specified by Eq*. (2), *such that*:

6 $$|\,{{\mbox{Tr}}}\,[\,{\rho }^{{\prime} }({{{{{{{\boldsymbol{x}}}}}}}}){O}_{{{{{{{{\boldsymbol{\theta }}}}}}}}}^{{\prime} }]-\,{{\mbox{Tr}}}\,[\,{\rho }_{{{{{{{{\boldsymbol{\theta }}}}}}}}}({{{{{{{\boldsymbol{x}}}}}}}}){O}_{{{{{{{{\boldsymbol{\theta }}}}}}}}}]|\le \,\delta,\,\forall {{{{{{{\boldsymbol{x}}}}}}}},{{{{{{{\boldsymbol{\theta }}}}}}}}.$$

*D* *the number of encoding gates used by the data re-uploading model and* ${\left|O\right|}_{\infty }$ *the spectral norm of its observable, the explicit model uses* ${{{{{{{\mathcal{O}}}}}}}}(D\log (D{\left|O\right|}_{\infty }{\delta }^{-1}))$ *additional qubits and gates.*

The general idea behind this construction is to encode the input data ***x*** in ancilla qubits, to finite precision, which can then be used repeatedly to approximate data-encoding gates using data-independent unitaries. More precisely, all data components ${x}_{i}\in {\mathbb{R}}$ of an input vector ***x*** = (*x*_1_, …, *x*_*d*_) are encoded as bit-strings $\left|{\widetilde{{{{{{{{\boldsymbol{x}}}}}}}}}}_{i}\right\rangle=|{b}_{0}{b}_{1}\ldots {b}_{p-1}\rangle \in {\{0,1\}}^{p}$, to some precision *ε* = 2^−*p*^ (e.g., using *R*_*x*_(*b*_*j*_) rotations on $\left|0\right\rangle$ states). Now, using *p* fixed rotations, e.g., of the form *R*_*z*_(2^−*j*^), controlled by the bits $|{b}_{j}\rangle$ and acting on *n* “working” qubits, one can encode every *x*_*i*_ in arbitrary (multi-qubit) rotations ${e}^{-{{{{{{{\rm{i}}}}}}}}{x}_{i}H}$, e.g., *R*_*z*_(*x*_*i*_), arbitrarily many times. Given that all these fixed rotations are data-independent, the feature encoding of any such circuit hence reduces to the encoding of the classical bit-strings ${\widetilde{{{{{{{{\boldsymbol{x}}}}}}}}}}_{i}$, prior to all variational operations. By preserving the variational unitaries appearing in a data re-uploading circuit and replacing its encoding gates with such controlled rotations, we can then approximate any data re-uploading model of the form of Eq. (5). The approximation error *δ* of this mapping originates from the finite precision *ε* of encoding ***x***, which results in an imperfect implementation of the encoding gates in the original circuit. But as *ε* → 0, we also have *δ* → 0, and the scaling of *ε* (or the number of ancillas *dp*) as a function of *δ* is detailed in Supplementary Section 2.

**Fig. 4:**
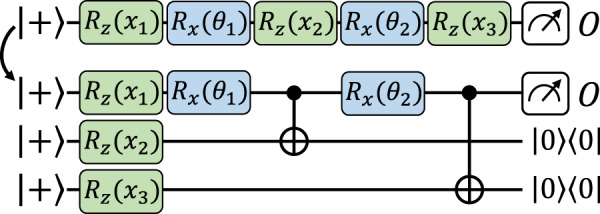
An exact mapping from a data re-uploading model to an equivalent explicit model, using gate teleportation. The details of this mapping, as well as its more elaborate form (using nested gate teleportation), can be found in Supplementary Section 2.

We now move to our main construction, resulting in exact mappings between data re-uploading and explicit models, i.e., that achieve *δ* = 0 with finite resources. We rely here on a similar idea to our previous construction, in which we encode the input data on ancilla qubits and later use data-independent operations to implement the encoding gates on the working qubits. The difference here is that we use gate-teleportation techniques, a form of measurement-based quantum computation^37^, to directly implement the encoding gates on ancillary qubits and teleport them back (via entangled measurements) onto the working qubits when needed (see Fig. 4).

**Theorem 1** *Given an arbitrary data re-uploading model* *f*_***θ***_(***x***) = Tr[*ρ*_***θ***_(***x***)*O*_***θ***_] *as specified by Eq*. (5), *there exists a mapping that produces an equivalent explicit model* ${\tilde{f}}_{{{{{{{{\boldsymbol{\theta }}}}}}}}}({{{{{{{\boldsymbol{x}}}}}}}})=\,{{\mbox{Tr}}}\,[{\rho }^{{\prime} }({{{{{{{\boldsymbol{x}}}}}}}}){O}_{{{{{{{{\boldsymbol{\theta }}}}}}}}}^{{\prime} }]$ *as specified by Eq*. (2), *such that*:

7 $$\,{{\mbox{Tr}}}\,[{\rho }^{{\prime} }({{{{{{{\boldsymbol{x}}}}}}}}){O}_{{{{{{{{\boldsymbol{\theta }}}}}}}}}^{{\prime} }]=\,{{\mbox{Tr}}}\,[{\rho }_{{{{{{{{\boldsymbol{\theta }}}}}}}}}({{{{{{{\boldsymbol{x}}}}}}}}){O}_{{{{{{{{\boldsymbol{\theta }}}}}}}}}],\,\forall {{{{{{{\boldsymbol{x}}}}}}}},{{{{{{{\boldsymbol{\theta }}}}}}}}.$$

*and* ${\left|{O}_{{{{{{{{\boldsymbol{\theta }}}}}}}}}^{{\prime} }\right|}_{\infty }^{2}\,\le \,{(1-{\delta }^{{\prime} })}^{-1}{\left|{O}_{{{{{{{{\boldsymbol{\theta }}}}}}}}}\right|}_{\infty }^{2}$, *for an arbitrary re-normalization parameter* ${\delta }^{{\prime} } \, > \, 0$. *For D the number of encoding gates used by the data re-uploading model*, *the equivalent explicit model uses* ${{{{{{{\mathcal{O}}}}}}}}(D\log (D/{\delta }^{{\prime} }))$ *additional qubits and gates*.

As we detail in Supplementary Section 2, gate teleportation cannot succeed with unit probability without gate-dependent (and hence data-dependent) corrections conditioned on the measurement outcomes of the ancilla. But since we only care about equality in expectation values (Tr[*ρ*_***θ***_(***x***)*O*_***θ***_] and $\,{{\mbox{Tr}}}\,[{\rho }^{{\prime} }({{{{{{{\boldsymbol{x}}}}}}}}){O}_{{{{{{{{\boldsymbol{\theta }}}}}}}}}^{{\prime} }]$), we can simply discard these measurement outcomes in the observable ${O}_{{{{{{{{\boldsymbol{\theta }}}}}}}}}^{{\prime} }$ (i.e., project on the correction-free measurement outcomes). In general, this leads to an observable with a spectral norm ${\left|{O}_{{{{{{{{\boldsymbol{\theta }}}}}}}}}^{{\prime} }\right|}_{\infty }^{2}={2}^{D}{\left|{O}_{{{{{{{{\boldsymbol{\theta }}}}}}}}}\right|}_{\infty }^{2}$ exponentially larger than originally, and hence a model that is exponentially harder to evaluate to the same precision. Using a nested gate-teleportation scheme (see Supplementary Section 2) with repeated applications of the encoding gates, we can however efficiently make this norm overhead arbitrarily small.

As our findings indicate, mappings from data re-uploading to explicit models are not unique, and seem to always incur the use of additional qubits. When discussing our learning separation results (see Corollary 1 below), we prove that this is indeed the case, and that any mapping from an arbitrary data re-uploading model with *D* encoding gates to an equivalent explicit model must use Ω(*D*) additional qubits in general. This makes our gate-teleportation mapping essentially optimal (i.e., up to logarithmic factors) in this extra cost.

To summarize, in this section, we demonstrated that linear quantum models can describe not only explicit and implicit models, but also data re-uploading circuits. More specifically, we showed that any hypothesis class of data re-uploading models can be mapped to an equivalent class of explicit models, that is, linear models with a restricted family of observables. In Supplementary Section 3, we extend this result and show that explicit models can also approximate any computable (classical or quantum) hypothesis class.

### Outperforming kernel methods with explicit and data re-uploading models

From the standpoint of relating quantum models to each other, we have shown that the framework of linear quantum models allows us to unify all standard models based on parametrized quantum circuits. While these findings are interesting from a theoretical perspective, they do not reveal how these models compare in practice. In particular, we would like to understand the advantages of using a certain model rather than the other in order to solve a given learning task. In this section, we address this question from several perspectives. First, we revisit the comparison between explicit and implicit models and clarify the implications of the representer theorem on the performance guarantees of these models. Then, we derive lower bounds for all three quantum models studied in this work in terms of their resource requirements, and show the existence of exponential separations between each pair of models. Finally, we discuss the implications of these results on the search for a quantum advantage in machine learning.

### Classical background and the representer theorem

Interestingly, a piece of functional analysis from learning theory gives us a way of characterizing any family of linear quantum models^25^. Namely, the so-called reproducing kernel Hilbert space, or RKHS^22^, is the Hilbert space ${{{{{{{\mathcal{H}}}}}}}}$ spanned by all functions of the form $f({{{{{{{\boldsymbol{x}}}}}}}})={\langle \phi ({{{{{{{\boldsymbol{x}}}}}}}}),w\rangle }_{{{{{{{{\mathcal{F}}}}}}}}}$, for all $w\in {{{{{{{\mathcal{F}}}}}}}}$. It includes any explicit and implicit models defined by the quantum feature states *ϕ*(***x***) = *ρ*(***x***). From this point of view, a relaxation of any learning task using implicit or explicit models as a hypothesis family consists in finding the function in the RKHS ${{{{{{{\mathcal{H}}}}}}}}$ that has optimal learning performance. For the supervised learning task of modeling a target function *g*(***x***) using a training set $\{\left({{{{{{{{\boldsymbol{x}}}}}}}}}^{(1)}\right.,g({{{{{{{{\boldsymbol{x}}}}}}}}}^{(1)}),\ldots,\left({{{{{{{{\boldsymbol{x}}}}}}}}}^{(M)},g({{{{{{{{\boldsymbol{x}}}}}}}}}^{(M)})\right.\}$, this learning performance is usually measured in terms of a training loss of the form, e.g.,

8 $$\widehat{{{{{{{{\mathcal{L}}}}}}}}}(\,\,f\,)=\frac{1}{M}\mathop{\sum }\limits_{m=1}^{M}{\left(f({{{{{{{{\boldsymbol{x}}}}}}}}}^{(m)})-g({{{{{{{{\boldsymbol{x}}}}}}}}}^{(m)})\right)}^{2}.$$

The true figure of merit of this problem, however, is in minimizing the expected loss ${{{{{{{\mathcal{L}}}}}}}}(f)$, defined similarly as a probability-weighted average over the entire data space ${{{{{{{\mathcal{X}}}}}}}}$. For this reason, a so-called regularization term $\lambda {\left|f\right|}_{{{{{{{{\mathcal{H}}}}}}}}}^{2}=\lambda {\left|O\right|}_{{{{{{{{\mathcal{F}}}}}}}}}^{2}$ is often added to the training loss ${\widehat{{{{{{{{\mathcal{L}}}}}}}}}}_{\lambda }(f)=\widehat{{{{{{{{\mathcal{L}}}}}}}}}(f)+\lambda {\left|O\right|}_{{{{{{{{\mathcal{F}}}}}}}}}^{2}$ to incentivize the model not to overfit on the training data. Here, *λ* ≥ 0 is a hyperparameter that controls the strength of this regularization.

Learning theory also allows us to characterize the linear models in ${{{{{{{\mathcal{H}}}}}}}}$ that are optimal with respect to the regularized training loss ${\widehat{{{{{{{{\mathcal{L}}}}}}}}}}_{\lambda }(f)$, for any *λ* ≥ 0. Specifically, the representer theorem^22^ states that the model ${f}_{{{{{\rm{opt}}}}}}\in {{{{{{{\mathcal{H}}}}}}}}$ minimizing ${\widehat{{{{{{{{\mathcal{L}}}}}}}}}}_{\lambda }(f)$ is always a kernel model of the form of Eq. (3) (see Supplementary Section 1 for a formal statement). A direct corollary of this result is that implicit quantum models are guaranteed to achieve a lower (or equal) regularized training loss than any explicit quantum model using the same feature encoding^25^. Moreover, the optimal weights *α*_*m*_ of this model can be computed efficiently using ${{{{{{{\mathcal{O}}}}}}}}({M}^{2})$ evaluations of inner products on a quantum computer (that is, by estimating the expectation value in Fig. 1b for all pairs of training points) and with classical post-processing in time ${{{{{{{\mathcal{O}}}}}}}}({M}^{3})$ using, e.g., ridge regression or support vector machines^22^. For this work, we ignore the required precision for the estimations of the quantum kernel. We note however that these can require exponentially many measurements in the number of qubits, both for explicit^38^ and implicit^27^ models.

This result may be construed to suggest that, in our study of quantum machine learning models, we only need to worry about implicit models, where the only real question to ask is what feature encoding circuit we use to compute a kernel function, and all machine learning is otherwise classical. In the next subsections, we show however the value of explicit and data re-uploading approaches in terms of generalization performance and resource requirements.

### Explicit can outperform implicit models

We turn our attention back to the explicit models resulting from our approximate mappings (see Fig. 3). Note that the kernel function associated with their bit-string encodings $\left|\psi ({{{{{{{\boldsymbol{x}}}}}}}})\right\rangle={\left|0\right\rangle }^{\otimes n}\left|\tilde{{{{{{{{\boldsymbol{x}}}}}}}}}\right\rangle$, $\rho ({{{{{{{\boldsymbol{x}}}}}}}})=\left|\psi ({{{{{{{\boldsymbol{x}}}}}}}})\right\rangle \left\langle \psi ({{{{{{{\boldsymbol{x}}}}}}}})\right|$, is trivially

9 $$k({{{{{{{\boldsymbol{x}}}}}}}},{{{{{{{{\boldsymbol{x}}}}}}}}}^{{\prime} })=\mathop{\prod }\limits_{i=1}^{d}{\left|\langle {\widetilde{{{{{{{{\boldsymbol{x}}}}}}}}}}_{i}|{\widetilde{{{{{{{{\boldsymbol{x}}}}}}}}}}_{i}^{{\prime} }\rangle \right|}^{2}={\delta }_{\widetilde{{{{{{{{\boldsymbol{x}}}}}}}}},{\widetilde{{{{{{{{\boldsymbol{x}}}}}}}}}}^{{\prime} }},$$

that is, the Kronecker delta function of the bit-strings $\widetilde{{{{{{{{\boldsymbol{x}}}}}}}}}$ and ${\widetilde{{{{{{{{\boldsymbol{x}}}}}}}}}}^{{\prime} }$. Let us emphasize that, for an appropriate precision *ε* of encoding input vectors ***x***, the family of explicit models resulting from our construction includes good approximations of virtually any parametrized quantum circuit model acting on *n* qubits. Yet, all of these result in the same kernel function of Eq. (9). This is a rather surprising result, for two reasons. First, this kernel is classically computable, which, in light of the representer theorem, seems to suggest that a simple classical model of the form of Eq. (3) can outperform any explicit quantum model stemming from our construction, and hence any quantum model in the limit *ε* → 0. Second, this implicit model always takes the form

10 $${f}_{{{{{{{{\boldsymbol{\alpha }}}}}}}},{{{{{{{\mathcal{D}}}}}}}}}({{{{{{{\boldsymbol{x}}}}}}}})=\mathop{\sum }\limits_{m=1}^{M}{\alpha }_{m}{\delta }_{\widetilde{{{{{{{{\boldsymbol{x}}}}}}}}},{\widetilde{{{{{{{{\boldsymbol{x}}}}}}}}}}^{(m)}},$$

which is a model that overfits the training data and fails to generalize to unseen data points, as, for *ε* → 0 and any choice of ***α***, ${f}_{{{{{{{{\boldsymbol{\alpha }}}}}}}},{{{{{{{\mathcal{D}}}}}}}}}({{{{{{{\boldsymbol{x}}}}}}}})=0$ for any ***x*** outside the training set. As we detail in Supplementary Section 2, similar observations can be made for the kernels resulting from our gate-teleportation construction.

These last remarks force us to rethink our interpretation of the representer theorem. When restricting our attention to the regularized training loss, implicit models do indeed lead to better training performance due to their increased expressivity. For example, on a classification task with labels *g*(***x***) = ±1, the kernel model of Eq. (10) is optimal with respect to any regularized training loss for *α*_*m*_ = *g*(***x***^(*m*)^) ∀ *m* such that $\widehat{{{{{{{{\mathcal{L}}}}}}}}}(\,f)=0$ and ${\left|f\right|}_{{{{{{{{\mathcal{H}}}}}}}}}^{2}=M$. But, as our construction shows, this expressivity can dramatically harm the generalization performance of the learning model, despite the use of regularization during training. Hence, restricting the set of observables accessible to a linear quantum model (or, equivalently, restricting the accessible manifold of the RKHS) can potentially provide a substantial learning advantage.

### Rigorous learning separations between all quantum models

Motivated by the previous illustrative example, we analyze more rigorously the advantages of explicit and data re-uploading models over implicit models. For this, we take a similar approach to recent works in classical machine learning which showed that neural networks can efficiently solve some learning tasks that linear or kernel methods cannot^39,40^. In our case, we quantify the efficiency of a quantum model in solving a learning task by the number of qubits and the size of the training set it requires to achieve a non-trivial expected loss. To obtain scaling separations, we consider a learning task specified by an arbitrary input dimension $d\in {\mathbb{N}}$ and express the resource requirements of the different quantum models as a function of *d*.

**Fig. 5:**
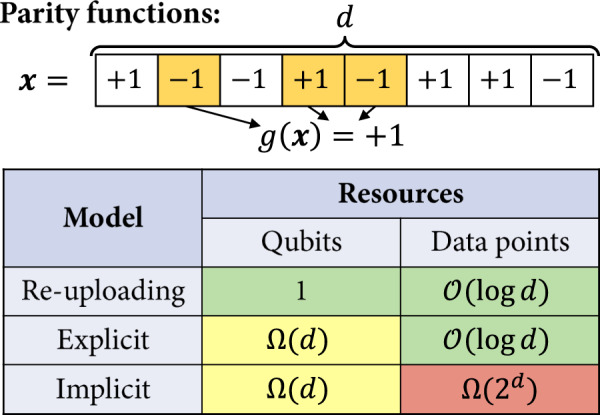
Learning separations. We describe a learning task based on parity functions acting on *d*-bit input vectors ***x*** ∈ {−1, 1}^*d*^, for $d\in {\mathbb{N}}$. This task allows us to separate all three quantum models studied in this work in terms of their resource requirements, as a function of *d* (see Theorem 2).

Similarly to ref. ^39^, the learning task we focus on is that of learning parity functions (see Fig. 5). These functions take as input a *d*-dimensional binary input ***x*** ∈ {−1, 1}^*d*^ and return the parity (i.e., the product) of a certain subset *A* ⊂ {1, …, *d*} of the components of ***x***. The interesting property of these functions is that, for any two choices of *A*, the resulting parity functions are orthogonal in the Hilbert space ${{{{{{{\mathcal{H}}}}}}}}$ of functions from {−1, 1}^*d*^ to ${\mathbb{R}}$. Hence, since the number of possible choices for *A* grow combinatorially with *d*, the subspace of ${{{{{{{\mathcal{H}}}}}}}}$ that these functions span also grows combinatorially with *d* (can be made into a 2^*d*^ scaling by restricting the choices of *A*). On the other hand, a linear model (explicit or implicit) also covers a restricted subspace (or manifold) of ${{{{{{{\mathcal{H}}}}}}}}$. The dimension of this subspace is upper bounded by 2^2*n*^ for a quantum linear model acting on *n* qubits, and by *M* for an implicit model using *M* training samples (see Supplementary Section 7 for detailed explanations). Hence, by essentially comparing these dimensions (2^*d*^ versus 2^2*n*^ and *M*)^40^, we can derive our lower bounds for explicit and implicit models. As for data re-uploading models, they do not suffer from these dimensionality arguments. The different components of ***x*** can be processed sequentially by the model, such that a single-qubit data re-uploading quantum circuit can represent (and learn) any parity function.

We summarize our results in the following theorem, and refer to Supplementary Section 7 for a more detailed exposition.

**Theorem 2** *There exists a regression task specified by an input dimension* $d\in {\mathbb{N}}$, *a function family* ${\{{g}_{A}:{\{-1,1\}}^{d}\to \{-1,1\}\}}_{A}$, *and associated input distributions* ${{{{{{{{\mathcal{D}}}}}}}}}_{A}$, *such that, to achieve an average mean-squared error*

$${{\mathbb{E}}}_{A}\left[\mathop{\inf }\limits_{f}{\left|f-{g}_{A}\right|}_{{L}^{2}({{{{{{{{\mathcal{D}}}}}}}}}_{A})}^{2}\right]=\varepsilon \, < \,1/2$$

(i) *any linear quantum model needs to act on*
  $$n\,\ge \,{{\Omega }}(d+\log (1-2\varepsilon ))$$
  *qubits*,
(ii) *any implicit quantum model additionally requires*
  $$M\,\ge \,{{\Omega }}({2}^{d}(1-2\varepsilon ))$$
  *data samples, while*
(iii) *a data re-uploading model acting on a single qubit and using d encoding gates can be trained to achieve a perfect expected error with probability* 1 − *δ*, *using* $M={{{{{{{\mathcal{O}}}}}}}}(\log (\frac{d}{\delta }))$ *data samples*.

A direct corollary of this result is a lower bound on the number of additional qubits that a universal mapping from any data re-uploading model to equivalent explicit models must use:

**Corollary 1** *Any universal mapping that takes as input an arbitrary data re-uploading model* *f*_***θ***_ *with* *D* *encoding gates and maps it to an equivalent explicit model* ${\widetilde{f}}_{{{{{{{{\boldsymbol{\theta }}}}}}}}}$ *must produce models acting on* Ω(*D*) *additional qubits for worst-case inputs*.

Comparing this lower bound to the scaling of our gate-teleportation mapping (Theorem 1), we find that it is optimal up to logarithmic factors.

### Quantum advantage beyond kernel methods

A major challenge in quantum machine learning is showing that the quantum methods discussed in this work can achieve a learning advantage over (standard) classical methods. While some approaches to this problem focus on constructing learning tasks with separations based on complexity-theoretic assumptions^17,19^, other works try to assess empirically the type of learning problems where quantum models show an advantage over standard classical models^11,20^. In this line of research, Huang et al.^20^ propose looking into learning tasks where the target functions are themselves generated by (explicit) quantum models. Following similar observations to those made above about the learning performance guarantees of kernel methods, the authors also choose to assess the presence of quantum advantages by comparing the learning performance of standard classical models only to that of implicit quantum models (from the same family as the target explicit models). This restricted comparison led to the conclusion that, with the help of training data, classical machine learning models could be as powerful as quantum machine learning models, even in these tailored learning tasks.

Having discussed the limitations of kernel methods in the previous subsections, we revisit this type of numerical experiments, where we additionally evaluate the performance of explicit models on these types of tasks.

**Fig. 6:**
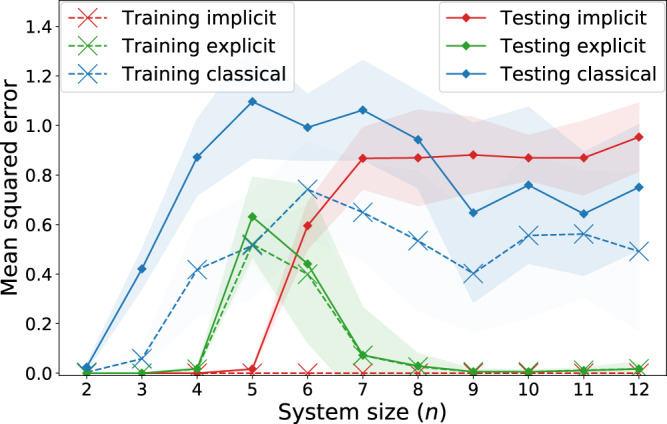
Regression performance of explicit, implicit and classical models on a “quantum-tailored” learning task. For all system sizes, each model has access to a training set of *M* = 1000 pre-processed and re-labeled fashion-MNIST images. Testing loss is computed on a test set of size 100. Shaded regions indicate the standard deviation over 10 labeling functions. The training errors of implicit models are close to 0 for all system sizes.

Similarly to Huang et al.^20^, we consider a regression task with input data from the fashion-MNIST dataset^41^, composed of 28 × 28-pixel images of clothing items. Using principal component analysis, we first reduce the dimension of these images to obtain *n*-dimensional vectors, for 2 ≤ *n* ≤ 12. We then label the images using an explicit model acting on *n* qubits. For this, we use the feature encoding proposed by Havlíček et al.^23^, which is conjectured to lead to classically intractable kernels, followed by a hardware-efficient variational unitary^4^. The expectation value of a Pauli *Z* observable on the first qubit then produces the data labels. Note that we additionally normalize the labels as to obtain a standard deviation of 1 for all system sizes. On this newly defined learning task, we test the performance of explicit models from the same function family as the explicit models generating the (training and test) data, and compare it to that of implicit models using the same feature encoding (hence from the same extended family of linear models), as well as a list of standard classical machine learning algorithms that are hyperparametrized for the task (see Supplementary Section 5). The results of this experiment are presented in Fig. 6.

The training losses we observe are consistent with our previous findings: the implicit models systematically achieve a lower training loss than their explicit counterparts. For an unregularized loss notably, the implicit models achieve a training loss of 0, and as noted in Supplementary Section 6, the addition of regularization to the training loss of the implicit model does not impact the separation we observe here. With respect to the testing loss on the other hand, which is representative of the expected loss, we see a clear separation starting from *n* = 7 qubits, where the classical models start having a competitive performance with the implicit models, while the explicit models clearly outperform them both. This goes to show that the existence of a quantum advantage should not be assessed only by comparing classical models to quantum kernel methods, as explicit (or data re-uploading) models can also conceal a substantially better learning performance.

## Discussion

In this work, we present a unifying framework for quantum machine learning models by expressing them as linear models in quantum feature spaces. In particular, we show how data re-uploading circuits can be represented exactly by explicit linear models in larger feature spaces. While this unifying formulation as linear models may suggest that all quantum machine learning models should be treated as kernel methods, we illustrate the advantages of variational quantum methods for machine learning. Going beyond the advantages in training performance guaranteed by the representer theorem, we first show how a systematic “kernelization" of linear quantum models can be harmful in terms of their generalization performance. Furthermore, we analyze the resource requirements (number of qubits and data samples used by) of these models, and show the existence of exponential separations between data re-uploading, linear, and kernel quantum models to solve certain learning tasks.

One takeaway message from our results is that training loss, even when regularized, is a misleading figure of merit. Generalization performance, which is measured on seen as well as unseen data, is in fact the important quantity to care about in (quantum) machine learning. These two sentences written outside of context will seem obvious to individuals well-versed in learning theory. However, it is crucial to recall this fact when evaluating the consequences of the representer theorem. This theorem only discusses regularized training loss, and thus despite its guarantees on the training loss of quantum kernel methods, it allows explicit models to have an exponential learning advantage in the number of data samples they use to achieve a good generalization performance.

From the limitations of quantum kernel methods highlighted by these results, we revisit a discussion on the power of quantum learning models relative to classical models in machine learning tasks with quantum-generated data. In a similar learning task to that of Huang et al.^20^, we show that, while standard classical models can be competitive with quantum kernel methods even in these “quantum-tailored” problems, variational quantum models can exhibit a significant learning advantage. These results give us a more comprehensive view of the quantum machine learning landscape and broaden our perspective on the type of models to use in order to achieve a practical learning advantage in the NISQ regime.

In this paper, we focus on the theoretical foundations of quantum machine learning models and how expressivity impacts generalization performance. But a major practical consideration is also that of trainability of these models. In fact, we know of obstacles in trainability for both explicit and implicit models. Explicit models can suffer from barren plateaus in their loss landscapes^38,42^, which manifest in exponentially vanishing gradients in the number of qubits used, while implicit models can suffer from exponentially vanishing kernel values^27,43^. While these phenomena can happen under different conditions, they both mean that an exponential number of circuit evaluations can be needed to train and make use of these models. Therefore, aside from the considerations made in this work, emphasis should also be placed on avoiding these obstacles to make good use of quantum machine learning models in practice.

The learning task we consider to show the existence of exponential learning separations between the different quantum models is based on parity functions, which is not a concept class of practical interest in machine learning. We note however that our lower bound results can also be extended to other learning tasks with concept classes of large dimensions (i.e., composed of many orthogonal functions). Quantum kernel methods will necessarily need a number of data points that scale linearly with this dimension, while, as we showcased in our results, the flexibility of data re-uploading circuits, as well as the restricted expressivity of explicit models can lead to substantial savings in resources. It remains an interesting research direction to explore how and when can these models be tailored to a machine learning task at hand, e.g., through the form of useful inductive biases (i.e., assumptions on the nature of the target functions) in their design.

## Supplementary information

Supplementary Information

## Data Availability

The data that support the plots within this paper are available at https://github.com/sjerbi/QML-beyond-kernel^44^. Source Data are provided with this paper.

## Source data

Source Data

## Code availability

The code used to run the numerical simulations, implemented using TensorFlow Quantum^45^, is available at https://github.com/sjerbi/QML-beyond-kernel^44^.
