# Supplementary material for: Quantum machine learning beyond kernel methods
Source: Nat Commun. 2023 Jan 31;14:517. doi: 10.1038/s41467-023-36159-y (PMC9889392; doi:10.1038/s41467-023-36159-y)
Supplement: Supplementary file 1 — Supplementary Information [file 41467_2023_36159_MOESM1_ESM.pdf]

Supplementary Information for: Quantum Machine Learning Beyond Kernel Methods

Sofiene Jerbi,[1] Lukas J. Fiderer,[1] Hendrik Poulsen Nautrup,[1] Jonas M. Kübler,[2] Hans J. Briegel,[1] and Vedran Dunjko[3]

[1] *Institute for Theoretical Physics, University of Innsbruck, Technikerstr. 21a, A-6020 Innsbruck, Austria*
[2] *Max Planck Institute for Intelligent Systems, Tübingen, Germany*
[3] *Leiden University, Niels Bohrweg 1, 2333 CA Leiden, Netherlands*

1. Representer theorem

In this supplementary section, we give a formal statement of the representer theorem from learning theory.

Theorem 1.1 (Representer theorem [1]). *Let $g : \mathcal{X} \to \mathcal{Y}$ be a target function with input and output domains $\mathcal{X}$ and $\mathcal{Y}$, $\mathcal{D} = \{(\boldsymbol{x}^{(1)}, g(\boldsymbol{x}^{(1)}), \ldots, (\boldsymbol{x}^{(M)}, g(\boldsymbol{x}^{(M)})\}$ a training set of size M, and $k : \mathcal{X} \times \mathcal{X} \to \mathbb{R}$ a kernel function with a corresponding reproducing kernel Hilbert space (RKHS) $\mathcal{H}$. For any strictly monotonic increasing regularization function $h : [0, \infty) \to \mathbb{R}$ and any training loss $\widehat{\mathcal{L}} : (\mathcal{X} \times \mathcal{Y})^M \times \mathcal{Y}^M \to \mathbb{R} \cup \infty$, we have that any minimizer of the regularized training loss from the RKHS $\mathcal{H}$,*

$$f_{\text{opt}} = \underset{f \in \mathcal{H}}{\operatorname{argmin}} \left\{ \widehat{\mathcal{L}}(\mathcal{D}, f(\mathcal{D}_{\boldsymbol{x}})) + h(\|f\|^2_{\mathcal{H}}) \right\}$$

admits a representation of the form

$$f_{\text{opt}}(\boldsymbol{x}) = \sum_{m=1}^{M} \alpha_m k(\boldsymbol{x}, \boldsymbol{x}^{(m)})$$

where $\alpha_m \in \mathbb{R}$ for all $1 \leq m \leq M$.

A common choice for the regularization function is simply $h(\|f\|^2_{\mathcal{H}}) = \lambda \|f\|^2_{\mathcal{H}}$, where $\lambda \geq 0$ is a hyperparameter adjusting the strength of the regularization.

2. Mappings from data re-uploading to explicit models

In this section, we detail possible mappings from data re-uploading models to explicit models, and prove Proposition 1 and Theorem 1 from the main text.

In our analysis, we restrict our attention to encoding gates of the form $e^{-\mathrm{i}h(\boldsymbol{x})H_n/2}$, for H_n an arbitrary Pauli string acting on n qubits, e.g., $H_3 = X \otimes Z \otimes I$, and $h : \mathbb{R}^d \to \mathbb{R}$ an arbitrary function mapping real-valued input vectors $\boldsymbol{x}$ to rotation angles $h(\boldsymbol{x})$. Using known techniques (see Sec. 4.7.3 in [2]), we can show that in order to implement any such gate $e^{-\mathrm{i}h(\boldsymbol{x})H_n/2}$ *exactly*, one only needs to perform a Pauli-Z rotation $e^{-\mathrm{i}h(\boldsymbol{x})Z/2}$ on a single of these n qubits, along with $\mathcal{O}(n)$ operations that are independent of $\boldsymbol{x}$ (and can therefore be absorbed by the surrounding variational unitaries). Therefore, in our mappings, we only need to focus on encoding gates of the form $R_z(h(\boldsymbol{x})) = e^{-\mathrm{i}h(\boldsymbol{x})Z/2}$.

A. Approximate bit-string mapping

We start by analyzing the resource requirements (in terms of number of additional qubits and gates) of our approximate bit-string mapping (Proposition 1).

Note that, in our construction (see Fig. 3 in the main text), the ancilla qubits are always prepared in computational basis states and only act as classical controls throughout the circuit. Hence, the operation $\mathrm{Tr}_{\widetilde{\boldsymbol{x}}}[V_1(\boldsymbol{\theta})C\text{-}\widetilde{U}_1 \ldots V_D(\boldsymbol{\theta})C\text{-}\widetilde{U}_D]$ obtained by tracing out these ancillas is equivalent to the *unitary* $V_1(\boldsymbol{\theta})U_1(\widetilde{x}_1)\ldots V_D(\boldsymbol{\theta})U_D(\widetilde{x}_D)$, where data-dependent rotations are only implemented to angle-precision $\varepsilon = 2^{-p}$. In the following, we relate this precision ε (or equivalently, the number of ancilla qubits dp)[1] to the approximation error $\delta \geq |\tilde{f}_{\boldsymbol{\theta}}(\boldsymbol{x}) - f_{\boldsymbol{\theta}}(\boldsymbol{x})|$ of our mapping.

Call $U = V_1(\boldsymbol{\theta})U_1(x_1)\ldots V_D(\boldsymbol{\theta})U_D(x_D)$ the data re-uploading unitary and $V = V_1(\boldsymbol{\theta})U_1(\widetilde{x}_1)\ldots V_D(\boldsymbol{\theta})U_D(\widetilde{x}_D)$ its approximation. We first relate the error δ to the distance measure $\|U - V\|_\infty = \max_{|\psi\rangle} \|(U-V)|\psi\rangle\|$:

$$\begin{aligned}
\left|\tilde{f}_{\boldsymbol{\theta}}(\boldsymbol{x}) - f_{\boldsymbol{\theta}}(\boldsymbol{x})\right| &= \left|\langle\psi| U^\dagger O U |\psi\rangle - \langle\psi| V^\dagger O V |\psi\rangle\right| \\
&= \left|\langle\psi| U^\dagger O |\Delta\rangle - \langle\Delta| O V |\psi\rangle\right| \\
&\leq \left|\langle\psi| U^\dagger O |\Delta\rangle\right| + \left|\langle\Delta| O V |\psi\rangle\right| \\
&\leq \sqrt{|\langle\psi| U^\dagger O^2 U |\psi\rangle|} \| |\Delta\rangle \| \\
&\quad + \sqrt{|\langle\psi| V^\dagger O^2 V |\psi\rangle|} \| |\Delta\rangle \| \\
&\leq 2\|O\|_\infty \|U - V\|_\infty
\end{aligned} \tag{1}$$

for $|\Delta\rangle = (U - V)|\psi\rangle$, by using the triangular and Cauchy-Schwarz inequalities to derive the first two inequalities, and the definition of the spectral norm $\|O\|_\infty$ and the distance $\|U - V\|_\infty$ to derive the last inequality.

Note that, for U and V obtained by sequences of unitary gates, the distance $\|U - V\|_\infty$ is linear in the pairwise distances between the gates in these sequences (see Sec. 4.5.3 of [2]):

$$\|U - V\|_\infty \leq \sum_{j=1}^{D} \|U_j - V_j\|_\infty \tag{2}$$

[1] We assume the worst-case scenario where each component x_i of $\boldsymbol{x}$ is assigned to a unique encoding gate, such that $D = d$.

therefore, to obtain $|\tilde{f}_{\boldsymbol{\theta}}(\boldsymbol{x}) - f_{\boldsymbol{\theta}}(\boldsymbol{x})| \leq \delta$, it is sufficient to enforce:

$$\|U_j - V_j\|_\infty \leq \frac{\delta}{2D\|O\|_\infty} \tag{3}$$

for each and single encoding gate in the circuit.

Since we assumed that the encoding gates of the circuit take the form $U_j = R_z(x_j)$, we can bound $\|U_j - V_j\|_\infty$ as a function of the precision ε of encoding x_j as:

$$\begin{aligned}\|U_j - V_j\|_\infty &= \max_{|\psi\rangle} \|(R_z(x_j+\varepsilon) - R_z(x_j))\,|\psi\rangle\| \\ &= \left\|e^{ix_j}(1-e^{i\varepsilon})\,|1\rangle\right\| = \left|1-e^{i\varepsilon}\right| \\ &\leq \sqrt{2}\varepsilon \end{aligned} \tag{4}$$

From Eqs. (3) and (4), we then get $\varepsilon \leq \frac{\delta}{2\sqrt{2}D\|O\|_\infty}$, or equivalently, $p \geq \lceil \log_2(\frac{2\sqrt{2}D\|O\|_\infty}{\delta})\rceil$. The number of additional qubits in the circuit is then Dp, which is also the number of additional gates (R_x data-encoding rotations and controlled data-independent rotations).

B. Mappings based on gate teleportation

We now move to our gate-teleportation mappings (Theorem 1). Again, we restrict our attention to teleporting $R_z(x)$ gates. This gate teleportation can easily be implemented using the gadget depicted in Supplementary Figure 1. It is easy to check that for an arbitrary input qubit $|\psi\rangle = \alpha\,|0\rangle + \beta\,|1\rangle$ and $|+\rangle = \frac{1}{\sqrt{2}}(|0\rangle + |1\rangle)$, the state generated by this gadget before the computational basis measurement (and correction) is:

$$\begin{aligned}&\frac{1}{\sqrt{2}}(\alpha\,|0\rangle + \beta e^{\mathrm{i}x}\,|1\rangle)\otimes|0\rangle + \frac{1}{\sqrt{2}}(\alpha e^{\mathrm{i}x}\,|0\rangle + \beta\,|1\rangle)\otimes|1\rangle \\ &= \frac{1}{\sqrt{2}}\left(R_z(x)\,|\psi\rangle\otimes|0\rangle + e^{\mathrm{i}x}R_z(-x)\,|\psi\rangle\otimes|1\rangle\right) \end{aligned} \tag{5}$$

which results in the correct outcome $|\psi'\rangle = R_z(x)\,|\psi\rangle$ for a $|0\rangle$ measurement, and a state that can be corrected (up to a global phase) via a $R_z(2x)$ rotation, in case of a $|1\rangle$ measurement.

Putting aside the corrections required by this gadget, we note the interesting property that, when used to simulate every encoding gate in the data re-uploading circuit, this gadget moves all data-dependent parts of the circuit on additional ancilla qubits, essentially turning it into an explicit model. However, this gadget still requires data-dependent corrections (of the form $R_z(2h(\boldsymbol{x}))$) in the case of $|1\rangle$ measurement outcomes, which happen with probability $1/2$ for each gate teleportation. To get around this problem, we simply replace the computational basis measurement in the gadget by a projection $P_0 = |0\rangle\langle 0|$ on the $|0\rangle$ state. While these projections cannot be implemented deterministically in practice, the resulting model is still a valid explicit model, in the sense that including the projections $P_0^{\otimes D}$ in the observable $O_{\boldsymbol{\theta}}$, for D uses of our gadget, still leads to a valid observable $O'_{\boldsymbol{\theta}} = O_{\boldsymbol{\theta}} \otimes P_0^{\otimes D}$.

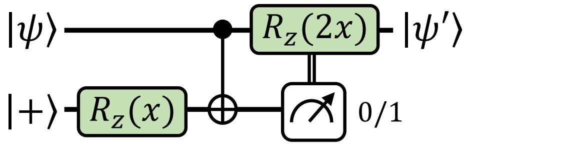

Supplementary Figure 1. A gate-teleportation gadget (inspired by the T-gadget in [3]) that implements the unitary $|\psi\rangle \mapsto |\psi'\rangle = R_z(x)\,|\psi\rangle$.

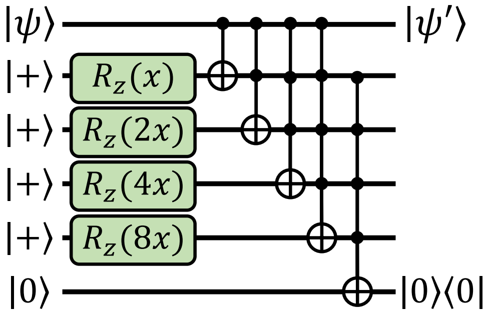

Supplementary Figure 2. A gate-teleportation gadget without data-dependent corrections and success probability $1 - 2^{-4} \approx 0.94$. The last qubit acts as a witness that at least one of the nested gate teleportations succeeded without the need for corrections.

However, given that each of these projections does not account for the re-normalization of the resulting quantum state (i.e., the factor $1/\sqrt{2}$ in Eq. (5)), this means that, in order to enforce $\mathrm{Tr}[\rho'(\boldsymbol{x})O'_{\boldsymbol{\theta}}] = \mathrm{Tr}[\rho_{\boldsymbol{\theta}}(\boldsymbol{x})O_{\boldsymbol{\theta}}]$, $\forall \boldsymbol{x}, \boldsymbol{\theta}$, we need to multiply $O'_{\boldsymbol{\theta}}$ by a factor of $2^{D/2}$. This implies that the evaluation of the resulting explicit model is exponentially harder than that of the original data re-uploading model, in the number of encoding gates D. As we show next, this factor can however be made arbitrarily close to 1, by allowing each encoding gate/angle to be used more than once in the feature encoding.

To achieve this feat, we transform our previous gadget as to implement its data-dependent corrections using gate teleportation again. A single such nested use of gate teleportation now has probability $1 - 1/4 = 3/4$ of succeeding without corrections, as opposed to the probability $1/2$ of the previous gadget. For N nested uses (see Supplementary Figure 2), the success probability is then boosted to $1 - 2^{-N}$, which can be made arbitrarily close to 1. If we use this nested gadget for all D encoding gates in the circuit, the probability that all of them are implemented successfully without corrections is then $p = (1 - 2^{-N})^D$. This probability p can be made larger than $1 - \delta'$, for any $\delta' > 0$, by choosing $N = \left\lceil \log_2\left((1 - \sqrt[D]{1-\delta'})^{-1}\right)\right\rceil \leq \lceil \log_2(D/\delta')\rceil$. This also leads to a normalization factor $\sqrt{p^{-1}} \leq \sqrt{(1-\delta')^{-1}}$, which can be made arbitrarily close to 1. Note as well that this normalization factor is always known exactly, such that we can guarantee $\mathrm{Tr}[\rho'(\boldsymbol{x})O'_{\boldsymbol{\theta}}] = \mathrm{Tr}[\rho(\boldsymbol{x},\boldsymbol{\theta})O_{\boldsymbol{\theta}}]$, $\forall \boldsymbol{x}, \boldsymbol{\theta}$.

C. Kernels resulting from our mappings

In the main text, we showed how our illustrative mapping based on bit-string encodings of $\boldsymbol{x}$ resulted in trivial Kronecker-delta kernel functions and implicit models with very poor generalization performance. In this subsection, we derive a similar result for our gate-teleportation mappings.

We first note that these mappings lead to feature encodings of the form:

$$U_\phi(\boldsymbol{x}) \left|0^{\otimes n+ND+D}\right\rangle = \left|0^{\otimes n+D}\right\rangle \bigotimes_{\substack{1\leq i\leq D\\ 1\leq j\leq N}} R_z(2^{j-1}h_i(\boldsymbol{x}))\left|+\right\rangle$$

for D encoding gates with encoding angles $h_i(\boldsymbol{x})$, and using N nested gate teleportations for each of these gates. While less generic than the feature states resulting from our binary encodings, these still generate kernels

$$k(\boldsymbol{x},\boldsymbol{x'}) = \prod_{i,j} \cos\!\left(2^{j-1}(h_i(\boldsymbol{x}) - h_i(\boldsymbol{x'}))\right)^2 \qquad (6)$$

that are again classically simulatable and $k(\boldsymbol{x},\boldsymbol{x'}) \to \delta_{\boldsymbol{x},\boldsymbol{x'}}$ for $ND \to \infty$.

D. Link to no-programming

It may seem to the informed reader as though our mappings from data re-uploading to explicit models are in violation of the so-called no-programming theorem from quantum information theory. In this section, we will shortly outline this theorem and explain why our mappings do not violate it.

A programmable quantum processor is defined as a CPTP map $\mathcal{C} : \mathcal{H}_\mathrm{S}\otimes\mathcal{H}_\mathrm{P} \to \mathcal{H}_\mathrm{S}$, where $\mathcal{H}_\mathrm{S}$ and $\mathcal{H}_\mathrm{P}$ denote the system and program Hilbert spaces. The purpose of such a quantum processor is to implement unitary maps $U : \mathcal{H}_\mathrm{S} \to \mathcal{H}_\mathrm{S}$ where the information about U is fed to the processor solely by a program state $|\psi_\mathrm{P}\rangle$ (see Supplementary Figure 3). The no-programming theorem [4, 5] rules out the existence of perfect universal quantum processors, in the sense that there cannot exist a processor $\mathcal{C}$ that can implement infinitely many different unitary maps U deterministically using finite-dimensional program states $|\psi_\mathrm{P}\rangle$.

The explicit models resulting from our mappings have properties that are reminiscent of quantum processors. In Fig. 3 in the main text for instance, the bit-string encodings $|\widetilde{\boldsymbol{x}}\rangle$ are used to implement data-encoding unitaries in an otherwise data-independent circuit. Thus, one may interpret these as the program states $|\psi(\boldsymbol{x})\rangle$ of a quantum processor $\mathcal{C}$ given by the rest of the circuit. Same goes for the quantum states $R_z(x_2)\left|+\right\rangle R_z(x_3)\left|+\right\rangle$ in Fig. 4 in the main text.

In light of the no-programming theorem, it is quite remarkable that these explicit models can "program" a *continuous* set of unitaries $\{U(\boldsymbol{x},\boldsymbol{\theta}) = \prod_\ell V_\ell(\boldsymbol{\theta})U_\ell(\boldsymbol{x})\}_{\boldsymbol{x}\in\mathbb{R}^d}$

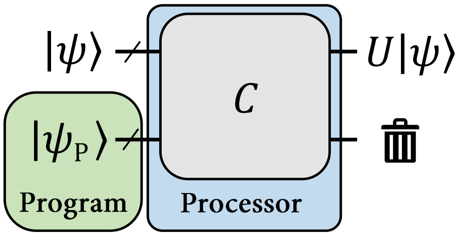

Supplementary Figure 3. A programmable quantum processor. A state $|\psi_\mathrm{P}\rangle$ is fed to the processor as a program instructing the processor to implement a unitary map U on another input state. The no-programming theorem states that programmable processors $\mathcal{C}$ capable of implementing any unitary map U cannot exist.

(and particularly the unitaries $U_\ell(\boldsymbol{x})$ for $\ell \geq 2$). Note however that, in the case of our bit-string mappings, these unitaries are only implemented *approximately* and that, in our gate-teleportation mappings, they are only implemented *probabilistically*. Our gate-teleportation mappings are only exact from the point of view of *models*, i.e., expectation values of observables, which are not covered by no-programming. The approximation errors $\delta > 0$ and the normalization factors $(1-\delta')^{-1} \neq 1$ that we obtain in our mappings are indeed symptomatic of our inability to program data re-uploading unitaries both exactly and deterministically. On the other hand, our results show that, contrary to unitary maps, expectation values can be "programmed" exactly.

3. Explicit models are universal function (family) approximators

From the universality of data re-uploading models as function approximators [6, 7] and our exact mappings from data re-uploading to explicit models, it trivially derives that explicit models are also universal function approximators. That is, for any integrable function $g \in \mathrm{L}([0,2\pi]^d)$ with a finite number of discontinuities, and for any $\varepsilon > 0$, there exists an n-qubit feature encoding of the form $U_\phi(\boldsymbol{x}) = I\bigotimes_{i,j} R_z(2^j x_i)H$ and an observable O such that $|\mathrm{Tr}[\rho(\boldsymbol{x})O] - g(\boldsymbol{x})| \leq \varepsilon, \forall \boldsymbol{x}$, for $\rho(\boldsymbol{x}) = U_\phi(\boldsymbol{x})\left|\boldsymbol{0}\middle\rangle\!\middle\langle\boldsymbol{0}\right| U_\phi^\dagger(\boldsymbol{x})$. This result derives specifically from Theorem 2 in [6], that we restate here for readability:

Theorem 3.1 (Adapted from Theorem 2 in [6])**.** *Consider any function $f : \mathbb{R} \to [0,1]$ with a finite number of finite discontinuities integrable within an interval $[a,b] \in \mathbb{R}$ of length P. Then, there exists a set of parameters $\boldsymbol{\theta}$ parametrizing a depth-$\mathcal{O}(N)$ 1-qubit data re-uploading circuit $U(\boldsymbol{x},\boldsymbol{\theta})$ such that*

$$\langle 1|\, U(\boldsymbol{x},\boldsymbol{\theta})\, |0\rangle = f_N(x), \qquad (7)$$

with $f_N(x)$ a Fourier series with N terms that satisfies $\lim_{N\to\infty} f_N(x) = f(x)$.

A similar result was independently obtained in Ref. [8].

In this section, we show that this universal approximation property of explicit models also applies to computable hypothesis classes, i.e., function families of a known classical or quantum model. More precisely, we show that for any family $\{g_{\boldsymbol{\theta}}\}_{\boldsymbol{\theta}}$ of *computable* functions $g_{\boldsymbol{\theta}} : [0, 2\pi]^d \to \mathbb{R}$ specified by a Boolean or quantum circuit parametrized by a vector $\boldsymbol{\theta} \in [0, 2\pi]^L$, and for any $\varepsilon > 0$, there exists an n-qubit feature encoding $U_\phi(\boldsymbol{x})$ using single-qubit rotations of the form $R_y(x_i)$ to encode $\boldsymbol{x}$, and a family of observables $O_{\boldsymbol{\theta}}$ parametrized by single-qubit rotations of the form $R_y(\theta_i)$, such that $|\mathrm{Tr}[\rho(\boldsymbol{x})O_{\boldsymbol{\theta}}] - g_{\boldsymbol{\theta}}(\boldsymbol{x})| \leq \varepsilon, \forall \boldsymbol{x}, \boldsymbol{\theta}$.

The proof of this result is rather simple. Using quantum amplitude (or phase) estimation [9] as a subroutine, we can, starting from a $|\mathbf{0}\rangle$ state and using $R_y(x_i)$ rotations, create a bit-string representation $|\widetilde{\boldsymbol{x}}\rangle$ of $\boldsymbol{x}$. This constitutes the feature encoding $U_\phi(\boldsymbol{x})$. On a different register, we create similarly a bit-string representation $|\widetilde{\boldsymbol{\theta}}\rangle$ of $\boldsymbol{\theta}$ using $R_y(\theta_i)$ rotations. These bit-strings, for an appropriately chosen precision of representation (which depends on the number of $R_y(x_i), R_y(\theta_i)$ rotations used), can then be used to approximate any computable function $g_{\boldsymbol{\theta}}(\boldsymbol{x})$ to some error ε.[2] Indeed, when $g_{\boldsymbol{\theta}}$ is computed via a parametrized quantum circuit, we can use a similar construction to that depicted in Fig. 3 in the main text. When $g_{\boldsymbol{\theta}}$ is instead computed classically (e.g., using a neural network), we can either simulate this computation with a quantum circuit (see Sec. 3.2.5 of [2]), or simply include it in the observables $O_{\boldsymbol{\theta}}$ as a post-processing of a computational basis measurement of $|\widetilde{\boldsymbol{x}}\rangle$ and $|\widetilde{\boldsymbol{\theta}}\rangle$.

The explicit models constructed in this proof may seem quite contrived and unnatural from the feature encodings and variational processing they use. Nonetheless, these constructions showcase how parametrized rotations, a natural building block to encode input data and to be used as variational gates, can leverage explicit quantum models to be universal function/model approximators.

4. Beyond unitary feature encodings

So far, in our definition of linear quantum models, we only considered *unitary* feature encodings, i.e., where feature states are defined as $\rho(\boldsymbol{x}) = U_\phi(\boldsymbol{x}) |\mathbf{0}\rangle\langle\mathbf{0}| U_\phi^\dagger(\boldsymbol{x})$ for a certain unitary map $U_\phi(\boldsymbol{x})$. In this section, we make the case that more general feature encodings, namely encodings for which the map $U_\phi(\boldsymbol{x})$ is allowed to be any completely positive trace preserving (CPTP) map, can lead to more interesting kernels $k(\boldsymbol{x}, \boldsymbol{x'}) = \mathrm{Tr}[\rho(\boldsymbol{x})\rho(\boldsymbol{x'})]$. This observation is in line with recent findings about quantum kernels derived from non-unitary feature encodings [10, 11].

We illustrate this point by focusing on the bit-string

[2] Note that, for functions $g_{\boldsymbol{\theta}}$ that are computed using binary representations of $\boldsymbol{x}$ and $\boldsymbol{\theta}$, this construction can be made exact.

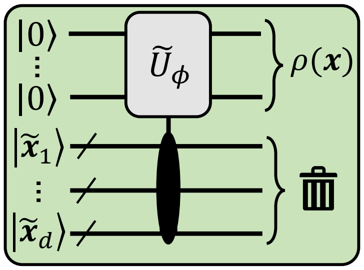

Supplementary Figure 4. A CPTP feature map based on bit-string encodings. Using data-independent controlled rotations, we can implement an approximation of any unitary feature encoding $U_\phi(\boldsymbol{x})$ by further tracing out the bit-string registers.

feature encoding $U_\phi(\boldsymbol{x}) \left|0^{\otimes n+dp}\right\rangle = |0^{\otimes n}\rangle \bigotimes_{i=1}^d |\widetilde{\boldsymbol{x}}_i\rangle$ that we presented in the main text. We start by noting that augmenting this feature encoding with an arbitrary unitary V always leads to the same kernel function:

$$k(\boldsymbol{x}, \boldsymbol{x'}) = \left| \langle \mathbf{0}| U_\phi^\dagger(\boldsymbol{x'}) V^\dagger V U_\phi(\boldsymbol{x}) |\mathbf{0}\rangle \right|^2 = \left| \langle \mathbf{0}| U_\phi^\dagger(\boldsymbol{x'}) U_\phi(\boldsymbol{x}) |\mathbf{0}\rangle \right|^2$$

given that $V^\dagger V = I$. If we however allow for a non-unitary operation such as tracing out part of the quantum system (which is allowed by CPTP maps), we can use this bit-string encoding to construct kernels $k(\boldsymbol{x}, \boldsymbol{x'})$ that approximate *virtually any* quantum kernel resulting from a unitary feature encoding on n qubits. To see this, suppose for instance that we want to approximate the quantum kernel proposed by Havlíček *et al.* [12] (resulting from the unitary feature encoding of Eq. (8)). In this case, we can, similarly to our construction in Fig. 3 in the main text, use data-independent rotations, controlled by the bit-string registers and acting on the n working qubits, to simulate the data-dependent gates of Eq. (8). Then, by tracing out the bit-string register, we effectively obtain an (arbitrarily good) approximation of the original feature encoding on the working qubits. This CPTP feature encoding is depicted in Supplementary Figure 4.

5. Details of the numerical simulations

In this section, we provide more details on the numerical simulations presented in the main text. We first describe how the training and testing data of the learning task are generated, then specify the quantum and classical models that we trained on this task.

A. Dataset generation

1. *Generating data points*

We generate our training and testing data by preprocessing the fashion MNIST dataset [13]. All 28 × 28-

pixels grayscale images in the dataset are first subject to a dimensionality reduction via principal component analysis (PCA), where only their n principal components are preserved, $2 \leq n \leq 12$. This PCA gives rise to data vectors $\boldsymbol{x} \in \mathbb{R}^n$ that are further normalized component-wise as to each be centered around 0 and have a standard deviation of 1. To create a training set, we sample $M = 1000$ of these vectors without replacement. A validation set and a test set, of size $M' = 100$ each, are sampled similarly from the pre-processed fashion MNIST testing data.

2. *Generating labels*

The labels $g(\boldsymbol{x})$ of the data points $\boldsymbol{x}$ in the training, validation and test sets are generated using the explicit models depicted in Supplementary Figure 5, for a number of qubits n equal to the dimension of $\boldsymbol{x}$, and uniformly random parameters $\boldsymbol{\theta} \in [0, 2\pi]^{3nL}$.
The feature encoding takes the form [12]:

$$U_\phi(\boldsymbol{x}) \left|0^{\otimes n}\right\rangle = U_z(\boldsymbol{x}) H^{\otimes n} U_z(\boldsymbol{x}) H^{\otimes n} \left|0^{\otimes n}\right\rangle \tag{8}$$

for

$$U_z(\boldsymbol{x}) = \exp\left(-\mathrm{i}\pi \left[\sum_{i=1}^{n} x_i Z_i + \sum_{\substack{j=1,\\ j>i}}^{n} x_i x_j Z_i Z_j\right]\right). \tag{9}$$

As for the variational unitaries $V(\boldsymbol{\theta})$, these are composed of L layers of single-qubit rotations $R(\boldsymbol{\theta}_{i,j})$ on each of the qubits, interlaid with $CZ = |1\rangle\langle 1| \otimes Z$ gates between nearest neighbours in the circuit. We choose the number of layers L as a function of the number of qubits n in the circuit, such that the number of parameters ($3nL$) is approximately 90 at all system sizes. Specifically, from $n = 2$ to 12, we have $L = 15, 10, 7, 6, 5, 4, 4, 3, 3, 3, 3$, respectively.
Finally, the labels of the data points are specified by the expectations values

$$g(\boldsymbol{x}) = w_{\mathcal{D},\boldsymbol{\theta}} \mathrm{Tr}[\rho(\boldsymbol{x}) V(\boldsymbol{\theta})^\dagger Z_1 V(\boldsymbol{\theta})] \tag{10}$$

where $w_{\mathcal{D},\boldsymbol{\theta}}$ is a re-normalization factor that sets the standard deviation of these labels to 1 over the training set.

3. *Evaluating performance*

We evaluate the training loss of a hypothesis function f using the mean squared error

$$\widehat{\mathcal{L}}(f) = \frac{1}{M} \sum_{m=1}^{M} \left(f(\boldsymbol{x}^{(m)}) - g(\boldsymbol{x}^{(m)})\right)^2 \tag{11}$$

on the training data. The test loss (indicative of the expected loss) is evaluated similarly on the test data (of size M').

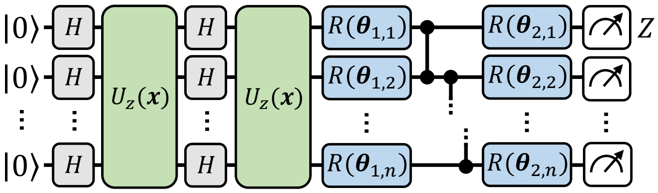

Supplementary Figure 5. The explicit model used in our numerical simulations. We use the feature encoding proposed by Havlíček *et al.* [12] (see Eq. (8)), followed by a hardware-efficient variational circuit, where arbitrary single-qubit rotations $R(\boldsymbol{\theta}_{i,j}) = R_x(\theta_{i,j,0}) R_y(\theta_{i,j,1}) R_z(\theta_{i,j,2})$ on each qubit are interlaid with nearest-neighbour CZ gates, for L layers (here $L = 2$). Finally, the expectation value of a Z_1 observable (with a re-normalization) assigns labels to input data $\boldsymbol{x} \in \mathbb{R}^n$.

B. Quantum machine learning models

In our simulations, we compare the performance of two types of quantum machine learning models.
First, we consider explicit models from the same variational family as those used to label the data (i.e., depicted in Supplementary Figure 5), but initialized with *different* variational parameters $\boldsymbol{\theta} \in [0, 2\pi]^{3nL}$, now sampled according to a independent normal distributions $\mathcal{N}(0, 0.05)$. As opposed to the generating functions of Eq. (10), we replace the observable weight $w_{\mathcal{D},\boldsymbol{\theta}}$ by a free parameter w, initialized to 1 and trained along the variational parameters $\boldsymbol{\theta}$. We train the explicit models for 500 steps of gradient descent on the training loss of Eq. (11). For this, we use an ADAM optimizer [14] with a learning rate $\alpha_{\boldsymbol{\theta}} = 0.01$ for the variational parameters $\boldsymbol{\theta}$ and a learning rate $\alpha_w = 0.1$ for the observable weight w.
Second, we also consider implicit models that rely on the same feature encoding $U_\phi(\boldsymbol{x})$ (Eq. (8)) as the explicit models. I.e., these take the form

$$f_{\boldsymbol{\alpha},\mathcal{D}}(\boldsymbol{x}) = \mathrm{Tr}[\rho(\boldsymbol{x}) O_{\boldsymbol{\alpha},\mathcal{D}}] \tag{12}$$

for the same encodings $\rho(\boldsymbol{x}) = U_\phi(\boldsymbol{x}) |\mathbf{0}\rangle\langle\mathbf{0}| U_\phi^\dagger(\boldsymbol{x})$, and an observable $O_{\boldsymbol{\alpha},\mathcal{D}}$ given by Eq. (4) in the main text. We train their parameters $\boldsymbol{\alpha}$ using the KernelRidge regression package of scikit-learn [15]. In the numerical simulations of Fig. 6 in the main text, we use an unregularized training loss, i.e., that of Eq. (11). The learning performance of the implicit models trained with regularization is presented in Supplementary Section 6.

C. Classical machine learning models

We additionally compare the performance of our quantum machine learning models to a list of classical models, identical to that of Huang *et al.* [11]. For completeness, we list these models here, and the hyperparameters they were trained with. All of these models were trained using the default specifications of scikit-learn [15], unless stated otherwise.

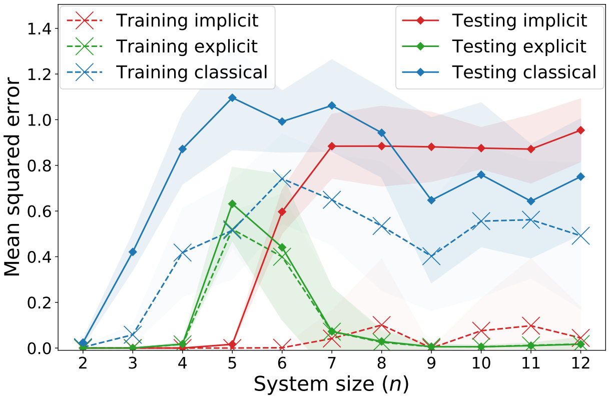

Supplementary Figure 6. Best performance of implicit models for different regularization strengths.

- Neural network: We perform a grid search over two-layer feed-forward neural networks with hidden layers of size

$$h \in \{10, 25, 50, 75, 100, 125, 150, 200\}.$$

We use the MLPRegressor package with a maximum number of learning steps max_iter = 500.

- Linear kernel method: We perform a grid search over the regularization parameter

$$C \in \{0.006, 0.015, 0.03, 0.0625, 0.125, 0.25, 0.5, 1.0, 2.0, 4.0, 8.0, 16.0, 32.0, 64.0, 128.0, 256, 512, 1024\}.$$

We select the best performance between the SVR and KernelRidge packages (both using the linear kernel).

- Gaussian kernel method: We perform a grid search over the regularization parameter

$$C \in \{0.006, 0.015, 0.03, 0.0625, 0.125, 0.25, 0.5, 1.0, 2.0, 4.0, 8.0, 16.0, 32.0, 64.0, 128.0, 256, 512, 1024\},$$

and the RBF kernel hyperparameter

$$\gamma \in \{0.25, 0.5, 1.0, 2.0, 3.0, 4.0, 5.0, 20.0\}/(n\mathrm{Var}[\boldsymbol{x}]),$$

where $\mathrm{Var}[\boldsymbol{x}]$ is the variance of all the components x_i, for all the data points $\boldsymbol{x}$ in the training set.
We select the best performance between the SVR and KernelRidge packages (both using the RBF kernel).

- Random forest: We perform a grid search over the individual tree depth

$$\text{max\_depth} \in \{2, 3, 4, 5\},$$

and the number of trees

$$\text{n\_trees} \in \{25, 50, 100, 200, 500\}.$$

We use the RandomForestRegressor package.

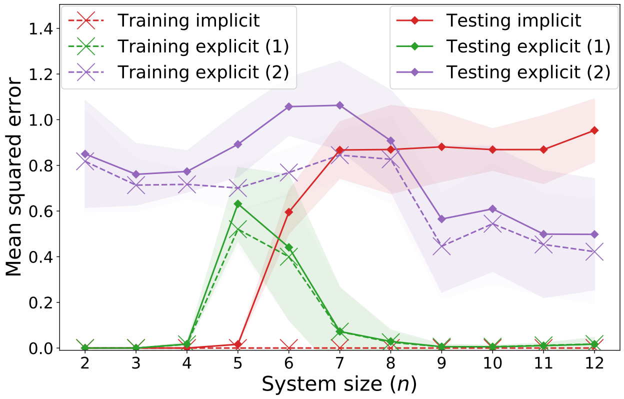

Supplementary Figure 7. Regression performance of explicit models from the same variational family as the models generating the data labels (1) and from a different variational family (2).

- Gradient boosting: We perform a grid search over the individual tree depth

$$\text{max\_depth} \in \{2, 3, 4, 5\},$$

and the number of trees

$$\text{n\_trees} \in \{25, 50, 100, 200, 500\}.$$

We use the GradientBoostingRegressor package.

- Adaboost: We perform a grid search over the number of estimators

$$\text{n\_estimators} \in \{25, 50, 100, 200, 500\}.$$

We use the AdaBoostRegressor package.

At each system size, we keep the learning performance of the model with the lowest validation loss and plot its test loss.

6. Additional numerical simulations

In this section, we defer the results of our additional numerical simulations, in support of the claims made in the main text.

We first show that regularization does not improve the learning performance of the implicit models. In Supplementary Figure 6 we plot the best test losses we obtained using regularization strengths $\lambda = 1/(2C)$ for $C \in \{0.006, 0.015, 0.03, 0.0625, 0.125, 0.25, 0.5, 1.0, 2.0, 4.0, 8.0, 16.0, 32.0, 64.0, 128.0, 256, 512, 1024\}$.

We also show that a variational family of observables not suited for the learning task can lead to poor learning performance. We illustrate this phenomenon by training explicit models constructed using the same feature encoding as those generating the data labels, but different variational unitaries $V(\boldsymbol{\theta})$ (see Eq. (10)). We take these

variational unitaries to resemble a Trotter evolution of a 1D-Heisenberg model with circular boundary conditions:

$$V(\boldsymbol{\theta}) = \prod_{i=1}^{L}\left(\prod_{j=1}^{n} e^{\mathrm{i}\theta_{i,j,0} Z_j Z_{j+1}} e^{\mathrm{i}\theta_{i,j,1} Y_j Y_{j+1}} e^{\mathrm{i}\theta_{i,j,2} X_j X_{j+1}}\right)$$

for the same number of layers L and the same number of parameters $\boldsymbol{\theta} \in [0, 2\pi]^{3nL}$ as the generating model. These are followed by a (weighted) Z_1 observable. Their learning performance is presented in Supplementary Figure 7.

7. Learning separations between quantum models

In this section, we establish rigorous learning separations between quantum learning models, using tools from recent works in classical machine learning [16, 17]. More specifically, we show the existence of a regression task specified by its input dimension $d \in \mathbb{N}$, such that, provably: (i) it can be solved exactly by a data re-uploading model acting on a single qubit and using $\mathcal{O}(\log(d))$ training samples, (ii) linear quantum models can also be sample efficient but require $\Omega(d)$ qubits to achieve a non-trivial expected loss, (iii) implicit models require both $\Omega(d)$ qubits and $\Omega(2^d)$ training samples to achieve this.

A. Learning parity functions

We consider the same learning task as Daniely & Malach [16], that is, learning k-sparse parity functions. These functions have discrete input and output spaces, $\mathcal{X} = \{-1, +1\}^d$ and $\mathcal{Y} = \{-1, +1\}$, respectively. $d \in \mathbb{N}$ specifies the dimension of the inputs $\boldsymbol{x} \in \mathcal{X}$, and an additional parameter $0 \leq k \leq d$ specifies the family of so-called k-sparse parity functions:

$$g_A(\boldsymbol{x}) = \prod_{i \in A} x_i$$

for $A \subset [d]$ and $|A| = k$. That is, for a given subset A of the input components $[d]$, the function g_A returns the parity ± 1 of these components for any input $\boldsymbol{x} \in \mathcal{X}$. A can be any subset of $[d]$ of size k, which gives us a family of functions $\{g_A\}_{A \subset [d], |A| = k}$ that we take to be our concept class (for a certain k specified later). These functions have the interesting property that they are all linearly independent (despite potentially being an exponentially large family), which is the essential property we'll be using to derive our separation results.

Daniely & Malach [16] show that, for an appropriate choice of input distribution and loss function, k-sparse parity functions cannot be approximated by any *polynomial-size* linear model (for $k \leq \frac{d}{16}$), while a depth-2 neural network with hidden layers polynomially large in k can learn these (almost) perfectly. The size of the linear model is defined as the dimension of its feature space $\mathcal{F}$ multiplied by the norm of its weight vector $\|w\|_{\mathcal{F}}$.

In our results, we rely instead on a powerful theorem by Hsu [17] to derive simpler separations results in terms of the *dimension* of linear models, rather than their size. This theorem, stated in the next subsection, makes it natural to consider an input distribution $\mathcal{D}_{\mathcal{X}}$ that is simply the uniform distribution over the data space $\mathcal{X}$, and the mean-squared error $\mathcal{L}_A(f) = \mathbb{E}_{\boldsymbol{x} \sim \mathcal{D}_{\mathcal{X}}}(f(\boldsymbol{x}) - g_A(\boldsymbol{x}))^2$ to be the expected loss for which we establish our bounds.

B. Learning performance of quantum models

This section is organized as follows: we first describe our lower bound results for linear quantum models, which are naturally derived for learning parities w.r.t. to the uniform input distribution $\mathcal{D}_{\mathcal{X}}$. We then move to our upper bound results for data re-uploading models, which make us consider a different input distribution $\mathcal{D}_A$ in order to achieve a largest separation with linear models. Together, these bounds give us our main Theorem 7.6.

1. *Lower bounds for linear models*

As mentioned above, our separation results derive from dimensionality arguments on the family of functions that can be represented by linear models. To start, let us consider the Hilbert space $\mathcal{H} = L^2(\mathcal{D}_{\mathcal{X}})$ of real-valued functions that are square-summable with respect to the probability space $(\mathcal{X}, 2^{\mathcal{X}}, \mathcal{D}_{\mathcal{X}})$. The inner product associated to this Hilbert space is $\langle f, g\rangle_{\mathcal{H}} = \frac{1}{|\mathcal{X}|}\sum_{\boldsymbol{x} \in \mathcal{X}} f(\boldsymbol{x}) g(\boldsymbol{x})$. For any $k \in \{0, \ldots, d\}$, k-sparse parity functions belong to $\mathcal{H}$, and they are moreover orthogonal functions of this Hilbert space. As for the functions generated by a linear model $f(\boldsymbol{x}) = \langle \phi(\boldsymbol{x}), w\rangle_{\mathcal{F}}$ (for a fixed feature encoding ϕ), these are also functions of the Hilbert space $\mathcal{H}$. More specifically, they are contained in a finite-dimensional subspace $\mathcal{W} = \operatorname{span}\{\langle \phi(\boldsymbol{x}), w_j\rangle_{\mathcal{F}}\}_{w_j}$ of $\mathcal{H}$, for $\{w_j\}_j$ a basis of $\mathcal{F}$ (with respect to the inner product $\langle \cdot, \cdot\rangle_{\mathcal{F}}$).
To establish our bounds, all we need to do now is: a) relate the dimension $\dim(\mathcal{W})$ of this subspace to the expected loss of the linear model, and b) upper bound $\dim(\mathcal{W})$ in terms of the number of qubits or data samples accessible to this model.

To get a), we use directly the following theorem:

Theorem 7.1 (Theorem 1 in [17], Theorem 29 in [18]). *Let $\varphi_1, \ldots, \varphi_N$ be orthogonal functions in a Hilbert space $\mathcal{H}$ such that $\|\varphi_i\|^2_{\mathcal{H}} = 1$ for all $i = 1, \ldots, N$, and let $\mathcal{W}$ be a finite subspace of $\mathcal{H}$. Then, for:*

$$\varepsilon = \frac{1}{N}\sum_{i=1}^{N}\left[\inf_{f \in \mathcal{W}} \|f - \varphi_i\|^2_{\mathcal{H}}\right],$$

we have

$$\dim(\mathcal{W}) \geq N(1 - \varepsilon).$$

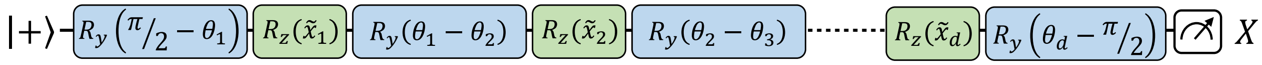

Supplementary Figure 8. Data re-uploading circuit used to learn parity functions. The encoding angles are $\widetilde{x}_i = \frac{\pi}{2}(x_i - 1)$, $\forall i = 1, \ldots, d$. Note that $R_y(\frac{\pi}{2})$ implements the transformation $|\pm\rangle \rightarrow |1/0\rangle$ and $R_y(-\frac{\pi}{2})$ implements $|1/0\rangle \rightarrow |\pm\rangle$, such that, to represent any parity function, we only need $\theta_i \in \{0, \frac{\pi}{2}\}, \forall i = 1, \ldots, d$.

By definition here, $\|f - \varphi_i\|_{\mathcal{H}}^2 = \frac{1}{|\mathcal{X}|} \sum_{\boldsymbol{x} \in \mathcal{X}} (f(\boldsymbol{x}) - \varphi_i(\boldsymbol{x}))^2$ is the mean-squared error $\mathcal{L}_i(f)$ of f with respect to φ_i. Given that, for $\{\varphi_i\}_{i \in [N]} = \{g_A\}_{A \subset [d], |A| = k}$, we have $N = \binom{d}{k}$, this theorem gives us a combinatorial lower bound on $\dim(\mathcal{W})$ when the linear model achieves a non-trivial average expected loss $\varepsilon < 1$ (otherwise obtained for $f(\boldsymbol{x}) = 0,\ \forall \boldsymbol{x} \in \mathcal{X}$).

We now move to point b). Note here that, in order to upper bound $\dim(\mathcal{W})$, all we need to do is upper bound the number of independent vectors in the $\operatorname{span}\{\langle \phi(\boldsymbol{x}), w_j \rangle_{\mathcal{F}}\}_{w_j} = \mathcal{W}$, hence, equivalently, the number of basis vectors w_j of $\mathcal{F}$. This can easily be done when we know the number of qubits on which the linear model acts. Indeed, for an n-qubit model, $\mathcal{F}$ is the space of $2^n \times 2^n$ Hermitian operators, and hence $\dim(\mathcal{W}) \leq 2^{2n}$. This leads us to our first lemma.

Lemma 7.2. *There exists a regression task specified by an input dimension $d \in \mathbb{N}$, a function family $\{g_A : \{-1,1\}^d \rightarrow \{-1,1\}\}_{A \subset [d], |A| = \lfloor d/2 \rfloor}$, and an input distribution $\mathcal{D}_{\mathcal{X}}$, such that, to achieve an average mean-squared error*

$$\mathbb{E}_A \big[\inf_{f \in \mathcal{W}} \|f - g_A\|_{L^2(\mathcal{D}_{\mathcal{X}})}^2 \big] \leq \varepsilon$$

any linear quantum model needs to act on

$$n \geq \frac{d}{4} + \frac{1}{2} \log_2(1 - \varepsilon)$$

qubits.

Proof. For $k = |A| = \lfloor \frac{d}{2} \rfloor$, we have $N = \binom{d}{k} \geq 2^{d/2}$. From Theorem 7.1, we have $\dim(\mathcal{W}) \geq 2^{d/2}(1 - \varepsilon)$, and from our previous observation, $\dim(\mathcal{W}) \leq 2^{2n}$. ☐

In the case of implicit linear models, note that we can bound $\dim(\mathcal{W})$ even more tightly. This is because the weight vector $w \in \mathcal{F}$, or equivalently the observable of the implicit quantum model, is expressed as a linear combination of embedded data samples $\phi(\boldsymbol{x}^{(i)}) = \rho(\boldsymbol{x}^{(i)})$. Therefore, the number of independent vectors in the $\operatorname{span}\{\langle \phi(\boldsymbol{x}), w_j \rangle_{\mathcal{F}}\}_{w_j} = \mathcal{W}$ is upper bounded by the number of data points M in the training set of the implicit model. This gives us $\dim(\mathcal{W}) \leq \min(2^{2n}, M)$ and the following lemma:

Lemma 7.3. *There exists a regression task specified by an input dimension $d \in \mathbb{N}$, a function family $\{g_A : \{-1,1\}^d \rightarrow \{-1,1\}\}_{A \subset [d], |A| = \lfloor d/2 \rfloor}$, and an input distribution $\mathcal{D}_{\mathcal{X}}$, such that, to achieve an average mean-squared error*

$$\mathbb{E}_A \big[\inf_{f \in \mathcal{W}} \|f - g_A\|_{L^2(\mathcal{D}_{\mathcal{X}})}^2 \big] \leq \varepsilon$$

any implicit quantum model needs to use

$$M \geq 2^{d/2}(1 - \varepsilon)$$

data samples.

Note that implicit quantum models suffer from both lower bounds in Lemmas 7.2 and 7.3: they require both $\Omega(d)$ qubits *and* $\Omega(2^d)$ data samples.

2. *Upper bound for data re-uploading models*

To establish our learning separation, we would like to show that data re-uploading circuits can efficiently represent parity functions. We show this constructively, by designing a single-qubit data re-uploading model that can compute any (k-sparse) parity function. This model is depicted in Supplementary Figure 8 and consists solely in $R_z(\widetilde{x}_i) = Z^{(x_i - 1)/2}$ encoding gates, parametrized R_y rotations and a final Pauli-X measurement. To understand how such a circuit can compute parity functions, consider the parity to be encoded in the qubit being either in a $|+\rangle$ or a $|-\rangle$ state. Therefore, the $R_z(\widetilde{x}_i)$ rotations flip the $|\pm\rangle$ state whenever $x_i = -1$, and preserve it otherwise. As for the $R_y(\pm\frac{\pi}{2})$ rotations, these essentially act as Hadamard gates by transforming a $|\pm\rangle$ state into a $|1/0\rangle$ state and back, which allows to "hide" this state from the action of a $R_z(\widetilde{x}_i)$ gate. We parametrize these gates as to hide the $|\pm\rangle$ qubit from $R_z(\widetilde{x}_i)$ whenever $\theta_i = 0$, and let it act whenever $\theta_i = \frac{\pi}{2}$. This leads us to the following lemma:

Lemma 7.4. *For the same learning task considered in Lemmas 7.2 and 7.3, there exists a data re-uploading model acting on a single qubit, and with depth $2d + 1$, that achieves a perfect mean-squared error:*

$$\mathbb{E}_A \big[\min_{f} \|f - g_A\|_{L^2(\mathcal{D}_{\mathcal{X}})}^2 \big] = 0.$$

Proof. For a given $A \subset [d]$, take in the circuit of Supplementary Figure 8: $\theta_i = \pi/2$ if $i \in A$ and $\theta_i = 0$ otherwise. ☐

Note that our claim on data re-uploading models so far only has to do with representability and not actual learning from data. We are yet to prove that a similar learning performance can be achieved from a training set of size polynomial in d and a polynomial-time learning algorithm. For the uniform data distribution $\mathcal{D}_{\mathcal{X}}$ we considered so far, this is known to be possible using $\mathcal{O}(d)$ data samples and by solving a resulting linear system of

equations acting on d variables [19]. However, this distribution does not provide us with the best possible separation in terms of data samples, which is why we consider instead the *mixture* of data distributions introduced by Daniely & Malach [16]. Originally intended to get around the hardness of learning parities with gradient-based algorithms [20], this distribution significantly reduces the data requirements for the data re-uploading (and explicit linear) models to $\mathcal{O}(\log(d))$, while preserving the $\Omega(2^d)$ lower bound for implicit models.

For every $A \subset [d]$, call $\mathcal{D}_A^{(1)} = \mathcal{D}_\mathcal{X}$ the uniform distribution over $\mathcal{X}$, and $\mathcal{D}_A^{(2)}$ the distribution where all components in $[d] \setminus A$ are drawn uniformly at random, while, independently, the components in A are all $+1$ with probability $1/2$ and all -1 otherwise. The data distribution $\mathcal{D}_A$ that we consider samples $\boldsymbol{x} \sim \mathcal{D}_A^{(1)}$ with probability $1/2$ and $\boldsymbol{x} \sim \mathcal{D}_A^{(2)}$ with probability $1/2$. For $k = |A|$ an odd number[3], this distribution is particularly interesting as, when $\boldsymbol{x} \sim \mathcal{D}_A^{(2)}$, $x_i = g_A(\boldsymbol{x})$ for all $i \in A$, which statistically "reveals" A, while $\mathcal{D}_A^{(1)}$ still preserves our previous hardness of generalization results. This allows us to prove the following lemma.

Lemma 7.5. *For the data distribution $\mathcal{D}_A$ defined above, there exists a learning algorithm using*

$$M = 32 \log\left(\frac{2d}{\delta}\right)$$

data samples and dM evaluations of the circuit in Supplementary Figure 8, that returns, for any $A \subset [d]$, with odd $|A| = k$, a function f_A satisfying

$$\|f_A - g_A\|^2_{L^2(\mathcal{D}_\mathcal{X})} = 0$$

with probability at least $1 - \delta$.

Proof. We analyze the following learning algorithm: given a training set of size M, evaluate, for all $i \in [d]$, the empirical loss $\widehat{\mathcal{L}}(f_i)$ of the data re-uploading function f_i obtained with the parameters $\theta_i = \frac{\pi}{2}$, $\theta_j = 0$ for $j \neq i$. Return $\theta_i = \frac{\pi}{2}$ when $\widehat{\mathcal{L}}(f_i) \leq 1.5$ and $\theta_i = 0$ otherwise, for all $i \in [d]$.

Call $X_i = (x_i - g_A(\boldsymbol{x}))^2$ the random variable obtained by sampling $\boldsymbol{x}$ from the data distribution $\mathcal{D}_A$, for a given A. Note that, by construction, $\mathbb{E}_{\mathcal{D}_A^{(1)}}[X_i] = 2$ and $\mathbb{E}_{\mathcal{D}_A^{(2)}}[X_i] = 0$ for $i \in A$, while $\mathbb{E}_{\mathcal{D}_A^{(1)}}[X_i] = \mathbb{E}_{\mathcal{D}_A^{(2)}}[X_i] = 2$ for $i \notin A$. Therefore $\mathbb{E}_{\mathcal{D}_A}[X_i] = 1$ for $i \in A$, and $\mathbb{E}_{\mathcal{D}_A}[X_i] = 2$ otherwise.

Given that the computed losses $\widehat{\mathcal{L}}(f_i)$ are empirical estimates of $\mathbb{E}_{\mathcal{D}_A}[X_i]$, all we need in order to identify A is guarantee with high probability that we can distinguish whether $\mathbb{E}_{\mathcal{D}_A}[X_i] = 1$ or 2, for all $i \in [d]$. We achieve this guarantee using the union bound and Hoeffding's inequality ($X_i \in [0, 4]$):

$$P\left(\bigcup_{i=1}^{d}\left(|\widehat{\mathcal{L}}(f_i) - \mathbb{E}[X_i]| \geq \frac{1}{2}\right)\right) \leq dP\left(|\widehat{\mathcal{L}}(f_i) - \mathbb{E}[X_i]| \geq \frac{1}{2}\right) \leq 2d \exp\left(-\frac{M}{32}\right)$$

and to upper bound this failure probability by δ, we need $M \geq 32 \log\left(\frac{2d}{\delta}\right)$. ☐

We leave as an open question whether an optimization procedure with similar learning guarantees but based on gradient descent also exists.

3. Main theorem

To conclude our results, we are left to show that similar lower bounds to those in Lemmas 7.2 and 7.3 also hold for the data distribution $\mathcal{D}_A$. Intuitively, this problem is easy to solve: given the inability of linear models to *represent* good approximations of parity functions with respect to the uniform data distribution, it should be clear that these still have a bad generalization performance with respect to $\mathcal{D}_A^{(1)}$, despite the information revealed by $\mathcal{D}_A^{(2)}$. We make this intuition rigorous in the following theorem (restatement of Theorem 2 in the main text).

Theorem 7.6. *There exists a regression task specified by an input dimension $d \in \mathbb{N}$, a function family $\{g_A : \{-1,1\}^d \to \{-1,1\}\}_{A\subset[d],|A|=\lfloor d/2\rfloor}$, and associated input distributions $\mathcal{D}_A$, such that, to achieve an average mean-squared error*

$$\mathbb{E}_A\left[\inf_{f\in\mathcal{W}} \|f - g_A\|^2_{L^2(\mathcal{D}_A)}\right] = \varepsilon < \frac{1}{2}$$

(i) any linear quantum model needs to act on

$$n \geq \frac{d}{4} + \frac{1}{2}\log_2(1 - 2\varepsilon)$$

qubits,
(ii) any implicit quantum model additionally requires

$$M \geq 2^{d/2}(1 - 2\varepsilon)$$

data samples, while
(iii) a data re-uploading model acting on a single qubit can be trained to achieve a perfect expected loss with probability $1 - \delta$, using $M = 32 \log\left(\frac{2d}{\delta}\right)$ data samples.

Proof. We relate ε to $\varepsilon_{A^{(1)}}$ (defined similarly to ε, but

[3] In Lemma 7.2, when $\lfloor \frac{d}{2} \rfloor$ is an even number, we can take $k = \lfloor \frac{d}{2} \rfloor + 1$, for which $\binom{d}{k} \geq 2^{d/2}$ still holds.

with respect to $\mathcal{D}_A^{(1)}$):

$$\begin{aligned}\varepsilon &= \mathbb{E}_A[\inf_{f\in\mathcal{W}} \|f - g_A\|^2_{L^2(\mathcal{D}_A)}] \\ &= \mathbb{E}_A[\inf_{f\in\mathcal{W}} \sum_{\boldsymbol{x}\in\mathcal{X}} \mathcal{D}_A(\boldsymbol{x})(f(\boldsymbol{x}) - g_A(\boldsymbol{x}))^2] \\ &\geq \frac{1}{2}\mathbb{E}_A[\inf_{f\in\mathcal{W}} \sum_{\boldsymbol{x}\in\mathcal{X}} \mathcal{D}_A^{(1)}(\boldsymbol{x})(f(\boldsymbol{x}) - g_A(\boldsymbol{x}))^2] \\ &\quad + \frac{1}{2}\mathbb{E}_A[\inf_{f\in\mathcal{W}} \sum_{\boldsymbol{x}\in\mathcal{X}} \mathcal{D}_A^{(2)}(\boldsymbol{x})(f(\boldsymbol{x}) - g_A(\boldsymbol{x}))^2] \\ &\geq \frac{1}{2}\varepsilon_{A^{(1)}}\end{aligned}$$

since $\varepsilon_{A^{(2)}} \geq 0$.
From Lemma 7.2, we have that, to ensure $\varepsilon_{A^{(1)}} \leq 2\varepsilon$, we need at least $\frac{d}{4} + \frac{1}{2}\log_2(1 - 2\varepsilon)$ qubits, which proves (i). (ii) follows similarly from Lemma 7.3.
Point (iii) corresponds to Lemmas 7.4 and 7.5. □

C. Tight bounds on linear realizations of data re-uploading models

A direct corollary of Lemmas 7.2 and 7.4 is a lower bound on the number of additional qubits required to map any data re-uploading model to an equivalent (explicit) linear model. Indeed, since the data re-uploading model of Supplementary Figure 8 can represent any parity function exactly for any input dimension $d \in \mathbb{N}$, while a linear model using a number of qubits sublinear in d can only achieve poor approximations on average, we can easily prove the following theorem (Corollary 1 in the main text).

Theorem 7.7. *Any procedure that takes as input an arbitrary data re-uploading model $f_{\boldsymbol{\theta}}$ with d encoding gates and returns an equivalent explicit model $\widetilde{f}_{\boldsymbol{\theta}}$ (i.e., a universal mapping) must produce models acting on $\Omega(d)$ additional qubits for worst-case inputs.*

Proof. By contradiction: were there a universal mapping using only $\widetilde{\mathcal{O}}(d^{1-\alpha})$, $\alpha > 0$ additional qubits, (i.e, sublinear in d), applying it to the circuit in Supplementary Figure 8 would result in a linear model acting on $\widetilde{\mathcal{O}}(d^{1-\alpha})$ qubits with perfect performance in representing parity functions, which contradicts Lemma 7.2. □

Note that our gate-teleportation mapping has an overhead of $\mathcal{O}(d\log(d/\delta'))$ qubits where δ' is a controllable parameter. This theorem hence proves that our mapping is *essentially* optimal with respect to this overhead.

D. The case of classification

So far, in our separation results, we have only considered a *regression* loss (the mean-squared error), despite the parity functions having a discrete output. It is an intriguing question whether similar separation results can be obtained in the case of *classification*, i.e., for a binary classification loss $\mathbb{E}_{\boldsymbol{x}\sim\mathcal{D}_{\mathcal{X}}}|\text{sign}(f(\boldsymbol{x}))-g_A(\boldsymbol{x})|$ for instance.
It is rather straightforward to show a $\Omega(d)$ qubits lower bound for linear classifiers that achieve *exact* learning (i.e., a 0 loss). We can consider here the concept class of all k-sparse parity functions for $k \in \{0,\ldots,d\}$, such that it contains all possible labelings $\mathcal{X} \to \{-1,1\}$. Therefore, a model that can represent all these functions exactly needs, by definition, a VC dimension larger than 2^d. But we know that, for linear quantum classifiers acting on n qubits, this VC dimension is upper bounded by $2^{2n}+1$ [21]. The lower bound then trivially derives.
Making this lower bound *robust* (i.e., allowing an $\varepsilon \geq 0$ loss) is however a harder task. By noting that a $\Omega(d)$ lower bound on the feature space dimension of a linear classifier implies a $\Omega(\log(d))$ lower bound on the number of qubits of a quantum linear classifier ($\dim(\mathcal{F}) = 2^{2n}$), we can adapt a result from Kamath *et al.* [22] to show the following: to achieve an average classification error ε on 1-sparse parity functions (i.e., $f_i(\boldsymbol{x}) = x_i$), a linear quantum classifier needs to act on $\Omega(\log[d(1 - H(\varepsilon))])$ qubits (for $H(\varepsilon)$ the binary entropy function). However, according to the same authors, establishing a stronger lower bound (e.g., number of qubits poly-logarithmic in d, or equivalently, a feature space dimension super-polynomial in d) for a similar task would constitute a major frontier in complexity theory. Such a result would provide with a function that requires a depth-2 threshold circuit of super-polynomial size to be computed, while the best known lower bounds for the size of depth-2 threshold circuit are only polynomial.

[1] B. Schölkopf, A. J. Smola, F. Bach, *et al.*, *Learning with kernels: support vector machines, regularization, optimization, and beyond* (MIT press, 2002).
[2] M. A. Nielsen and I. Chuang, *Quantum computation and quantum information* (American Association of Physics Teachers, 2002).
[3] S. Bravyi and D. Gosset, Improved classical simulation of quantum circuits dominated by clifford gates, Physical review letters **116**, 250501 (2016).
[4] M. A. Nielsen and I. L. Chuang, Programmable Quantum Gate Arrays, Physical Review Letters **79**, 321 (1997).
[5] Y. Yang, R. Renner, and G. Chiribella, Optimal Universal Programming of Unitary Gates, Physical Review Letters **125**, 210501 (2020).
[6] A. Pérez-Salinas, D. López-Núñez, A. García-Sáez, P. Forn-Díaz, and J. I. Latorre, One qubit as a universal approximant, Physical Review A **104**, 012405 (2021).
[7] M. Schuld, R. Sweke, and J. J. Meyer, Effect of data

encoding on the expressive power of variational quantum-machine-learning models, Physical Review A **103**, 032430 (2021).

[8] T. Goto, Q. H. Tran, and K. Nakajima, Universal approximation property of quantum machine learning models in quantum-enhanced feature spaces, Phys. Rev. Lett. **127**, 090506 (2021).

[9] G. Brassard, P. Hoyer, M. Mosca, and A. Tapp, Quantum amplitude amplification and estimation, Contemporary Mathematics **305**, 53 (2002).

[10] J. M. Kübler, S. Buchholz, and B. Schölkopf, The inductive bias of quantum kernels, Advances in Neural Information Processing Systems **34** (2021).

[11] H.-Y. Huang, M. Broughton, M. Mohseni, R. Babbush, S. Boixo, H. Neven, and J. R. McClean, Power of data in quantum machine learning, Nature communications **12**, 1 (2021).

[12] V. Havlíček, A. D. Córcoles, K. Temme, A. W. Harrow, A. Kandala, J. M. Chow, and J. M. Gambetta, Supervised learning with quantum-enhanced feature spaces, Nature **567**, 209 (2019).

[13] H. Xiao, K. Rasul, and R. Vollgraf, Fashion-mnist: a novel image dataset for benchmarking machine learning algorithms, arXiv:1708.07747 (2017).

[14] D. P. Kingma and J. Ba, Adam: A method for stochastic optimization, arXiv:1412.6980 (2014).

[15] F. Pedregosa, G. Varoquaux, A. Gramfort, V. Michel, B. Thirion, O. Grisel, M. Blondel, P. Prettenhofer, R. Weiss, V. Dubourg, *et al.*, Scikit-learn: Machine learning in python, the Journal of machine Learning research **12**, 2825 (2011).

[16] A. Daniely and E. Malach, Learning parities with neural networks, Advances in Neural Information Processing Systems **33** (2020).

[17] D. Hsu, Dimension lower bounds for linear approaches to function approximation, Daniel Hsu's homepage (2021).

[18] D. Hsu, C. H. Sanford, R. Servedio, and E. V. Vlatakis-Gkaragkounis, On the approximation power of two-layer networks of random relus, in *Proceedings of Thirty Fourth Conference on Learning Theory*, Proceedings of Machine Learning Research, Vol. 134, edited by M. Belkin and S. Kpotufe (PMLR, 2021) pp. 2423–2461.

[19] M. Kearns, Efficient noise-tolerant learning from statistical queries, Journal of the ACM (JACM) **45**, 983 (1998).

[20] S. Shalev-Shwartz, O. Shamir, and S. Shammah, Failures of gradient-based deep learning, in *International Conference on Machine Learning* (PMLR, 2017) pp. 3067–3075.

[21] C. Gyurik, D. van Vreumingen, and V. Dunjko, Structural risk minimization for quantum linear classifiers, arXiv:2105.05566 (2021).

[22] P. Kamath, O. Montasser, and N. Srebro, Approximate is good enough: Probabilistic variants of dimensional and margin complexity, in *Conference on Learning Theory* (PMLR, 2020) pp. 2236–2262.
